# Laser desorption rapid evaporative ionization mass spectrometry (LD-REIMS) demonstrates a direct impact of hypochlorous acid stress on PQS-mediated quorum sensing in *Pseudomonas aeruginosa*

**DOI:** 10.1128/msystems.01165-23

**Published:** 2024-03-26

**Authors:** Rob Bradley, Daniel Simon, Livia Spiga, Yuchen Xiang, Zoltan Takats, Huw Williams

**Affiliations:** 1 Department of Life Sciences, Faculty of Natural Sciences, Imperial College London, London, United Kingdom; 2 Department of Metabolism, Digestion and Reproduction, Faculty of Medicine, Imperial College London, London, United Kingdom; 3 The Rosalind Franklin Institute, Didcot, United Kingdom; Georgia Institute of Technology, Atlanta, Georgia, USA

**Keywords:** *Pseudomonas aeruginosa*, laser desorption rapid evaporative ionization mass spectrometry, oxidative stress, quorum sensing, hypochlorous acid, hypothiocyanous acid

## Abstract

To establish infections in human hosts, *Pseudomonas aeruginosa* must overcome innate immune-generated oxidative stress, such as the hypochlorous acid (HOCl) produced by neutrophils. We set out to find specific biomarkers of oxidative stress through the development of a protocol for the metabolic profiling of *P. aeruginosa* cultures grown in the presence of different oxidants using a novel ionization technique for mass spectrometry, laser desorption rapid evaporative ionization mass spectrometry (LD-REIMS). We demonstrated the ability of LD-REIMS to classify samples as untreated or treated with a specific oxidant with 100% accuracy and identified a panel of 54 metabolites with significantly altered concentrations after exposure to one or more of the oxidants. Key metabolic changes were conserved in *P. aeruginosa* clinical strains isolated from patients with cystic fibrosis lung infections. These data demonstrated that HOCl stress impacted the *Pseudomonas* quinolone signal (PQS) quorum sensing system. Ten 2-alkyl-4-quinolones (AHQs) associated with the PQS system were significantly lower in concentration in HOCl-stressed *P. aeruginosa* cultures, including 2-heptyl-3-hydroxy-4(1H)-quinolone (PQS), the most active signal molecule of the PQS system. The PQS system regulates the production of virulence factors, including pyocyanin and elastase, and their levels were markedly affected by HOCl stress. No pyocyanin was detectable and elastase concentrations were reduced by more than 75% in cultures grown with sub-lethal concentrations of HOCl, suggesting that this neutrophil-derived oxidant may disrupt the ability of *P. aeruginosa* to establish infections through interference with production of PQS-associated virulence factors.

**IMPORTANCE:**

This work demonstrates that a high-throughput ambient ionization mass spectrometry method can be used successfully to study a bacterial stress response. Its application to the opportunistic pathogen *Pseudomonas aeruginosa* led to the identification of specific oxidative stress biomarkers, and demonstrated that hypochlorous acid, an oxidant specifically produced by human neutrophils during infection, affects quorum sensing and reduces production of the virulence factors pyocyanin and elastase. No pyocyanin was detectable and elastase levels were reduced by more than 75% in bacteria grown in the presence of hypochlorous acid. This approach has the potential to be widely applicable to the characterization of the stress responses of bacteria.

## INTRODUCTION

Bacteria encounter oxidative stress in aerobic environments. The innate immune systems of both animals and plants use reactive oxygen species to attack and to attempt to kill invading microbes. Upon phagocytosis of bacteria, the phagocyte or NADPH oxidase of human neutrophils is induced, assembled, and catalyzes the formation of the superoxide radical (O_2_^.-^). This is known as the respiratory burst and is associated with a 10- to 20-fold increase in O_2_ consumption (1). The importance of superoxide generated by NADPH oxidase is demonstrated through the susceptibility of people with defective NADPH oxidase to infection by a broad range of microbes, as seen in conditions such as chronic granulomatous disease (CDG) (2). However, superoxide has low reactivity and is membrane impermeable, and so the antimicrobial action of the respiratory burst is thought to be mediated through the conversion of superoxide to stronger oxidants, including H_2_O_2_, hypochlorous acid (HOCl) and hypothiocyanous acid (HOSCN) (3). O_2_^.-^ dismutates either spontaneously or catalyzed by superoxide dismutase to form H_2_O_2_. HOCl and HOSCN can then be formed by the reaction of H_2_O_2_ with chloride or thiocyanate ions respectively, catalyzed by myeloperoxidase (MPO). While there exists a large body of evidence to indicate that H_2_O_2_ is important for the killing of pathogens by neutrophils (4)(5) the antimicrobial actions of HOCl and HOSCN are relatively poorly studied, despite MPO comprising around 5% of neutrophil proteins (1) and that neutrophil-mediated killing of bacteria has been shown to be MPO-dependent (6).

To further understand their bactericidal action and the corresponding bacterial protective response, we previously performed a genetic and transcriptomic analysis of the response of *Pseudomonas aeruginosa* to HOCl and HOSCN (7). We identified a diverse range of genes required for protection against HOCl and HOSCN, including the transcriptional regulator RclR, a homolog of the *Escherichia coli* HOCl-specific sensor RclR. We demonstrated that RclR regulates the expression of *rclX*, a putative peroxiredoxin, in *P. aeruginosa* and that both RclR and RclX are required specifically for protection against HOCl and HOSCN, with RclX shown to be a HOCl reductase (8). Another key study showed that hypohalous acids, such as HOCl, caused protein aggregation and that *P. aeruginosa* responded by increasing polyphosphate levels, which improved survival by protecting against protein aggregation (9).

We wanted to complement these studies by investigating the metabolic changes that occur following oxidative stress to provide additional mechanistic insights and to identify biomarkers of the stress to provide a way of examining bacterial stress exposure *in vivo* during infection. In parallel we were interested in establishing whether laser desorption rapid evaporative ionization mass spectrometry (LD-REIMS) can be used to study a bacterial stress response.

REIMS is a novel ionization method for mass spectrometry, originally developed as an intra-operative, *in situ* method for tumour margin detection, in which rapid heating of a sample generates an aerosol containing gas phase ions of metabolites and biological molecules that can be analyzed to provide real time, semi-quantitative metabolic profiling (10). Rapid heating can be achieved through the application of electrical diathermy, for example in the form of a heated electrosurgical knife, or by use of a laser, as in LD-REIMS. REIMS is an ambient ionization mass spectrometry (AIMS) technique, meaning it allows for mass spectrometry analysis of biological samples in their natural state (11). Established AIMS techniques such as desorption electrospray ionization (DESI) and direct analysis in real time (DART) have previously been successfully applied to microbial metabolomics, allowing for imaging of microbial interactions (12–14) and high throughput identification of bacteria (15–19). Based on these promising applications of AIMS techniques, REIMS has been expanded from a surgical tool to applications in microbiology. The Takats group used electrical diathermy REIMS to characterize the metabolomes of *P. aeruginosa* clinical isolates by analyzing colonies grown on agar plates, successfully classifying isolates to a strain level with 81% accuracy and identifying a number of *P. aeruginosa* metabolites (20), while LD-REIMS has been used successfully for metabolic fingerprinting of biofluids and for screening of libraries for synthetic biology applications, able to detect a panel of diverse biological molecules in a yeast model, demonstrating its potential for broad applicability to metabolite detection (21–25).

The aim of this study was to determine whether LD-REIMS can be used as a high-throughput approach to study bacterial stress responses. We wanted first to test whether LD-REIMS can distinguish untreated from oxidatively stressed *P. aeruginosa*, and subsequently whether it can be used to reliably identify changes in the oxidant-specific biomarkers in this bacterium, as well as to provide mechanistic insights into the response to oxidative stress.

## MATERIALS AND METHODS

### Bacterial strains and growth conditions

The *P. aeruginosa* lab strain PA14 was used for all experiments unless otherwise stated. The transposon mutant *pqsE* came from a *P. aeruginosa* transposon mutant library (26). The clinical isolates C1, C2, C3, C9, C13, C14, C25, C28, and C50 were supplied by the Royal Brompton Hospital (Table S1). All liquid cultures were grown in LB medium (5 g/L NaCl, 5 g/L yeast extract, and 10 g/L tryptone) at 37°C, shaking at 700 rpm.

### Preparation of oxidants for bacterial stress

#### Sodium hypochlorite

HOCl solutions were prepared by diluting NaOCl (Sigma) directly into the media. The molar concentration of OCl^−^ was determined using iodometric titration. Potassium iodide (KI) and hydrochloric acid (HCl) were added to NaOCl, and the resulting solution titrated to a colorless end-point using sodium thiosulphate (Na_2_S_2_O_3_) and a starch solution indicator.

#### Hypothiocyanous acid

HOSCN solutions were generated as described in reference (27) with modifications. Briefly, a solution containing 6.5 mM potassium thiocyanate (KSCN, Fluka) and 6 U/mL lactoperoxidase (LPO, Sigma) was prepared in LB. Aliquots of 1 mM H_2_O_2_ were added over 5 min, followed by the addition of 100 U/mL catalase (Sigma). To remove proteins, the solution was centrifuged at 4,000 rpm for 10 min using a 10-kDa filter (Ambion). The concentration of HOSCN was determined by monitoring the loss of signal at 412 nm (ε412 nm = 14,150 M^−1^ cm^−1^) that occurs when HOSCN reacts with 5-thio-2-nitrobenzoic acid (TNB) (28).

#### Hydrogen peroxide (H_2_O_2_) solutions

Solutions were prepared by diluting 30% wt/wt in H_2_O (9.79 M, Sigma) directly into the growth medium.

#### Methylglyoxal solutions

MGO solutions were prepared by diluting MGO (Sigma, 40% in H_2_O) directly into the growth medium.

#### Methyl viologen solutions

MV solutions were prepared by diluting MV (Sigma, 10 mM) directly into the growth medium.

### Preparation of liquid-culture grown bacterial samples for LD-REIMS analysis

LB medium was inoculated with *P. aeruginosa* in a 96-deep-well plate and cultured overnight at 37°C, shaking at 700 rpm on a THERMOstar microplate incubator and shaker. Each replicate was an independent culture. The overnight cultures were subcultured 1:50 into 190 µL of fresh media spiked with the oxidant of interest in a 96-well plate and incubated at 37°C, shaking at 700 rpm. Once balanced growth was achieved in the mid-exponential phase, the cultures were transferred to a V-shaped-bottom 96-well plate (Sigma) and centrifuged at 3,270 × *g* for 20 min at 4°C. The supernatant was removed, the pellets resuspended in 100 µL of sterile ammonium acetate solution (150 mM) and again centrifuged at 3720 × *g* for 20 min, at 4°C. The supernatant was removed, and the plate containing the bacterial cell pellet was introduced into the LD-REIMS plate reader for analysis.

### LD-REIMS analysis

LD-REIMS analysis was performed by adapting the methodology described by reference (29). The pellets were subject to laser ablation using an Opolette 2940 laser system (Opotek). One laser burn was performed per pellet, with a fluence of 5 J/cm^2^ and a laser firing duration of 3 s. Samples were analyzed at 3 s intervals. The aerosol produced was transported by vacuum through polytetrafluorethylene tubing (3.2 mm O.D., 1.6 mm I.D.) connected to the REIMS interface through a T-shaped piece, also permitting inflow of isopropanol (IPA) at a flow rate of 0.1 mL/min, as described by reference (30). All mass analysis was carried out in negative ionization mode using a Xevo G2-S QToF instrument with the cone voltage of 40 V, heater bias voltage of 80 V, and *m/z* scan range from 50 to 1,200 Da. The REIMS instrument was calibrated in sensitivity mode according to the manufacturer’s standard instructions (Waters Corporation), using 0.1 mM sodium formate solution in isopropanol and water (90/10, vol/vol) at a flow rate of 0.1 mL/min.

### Data processing and statistical analysis

Peak picking was performed using an in-house workflow based in R and Python and developed by members of the Takats group. The script is available on request from the authors. In summary, first, the raw data were background subtracted and lock mass corrected using one of three known *P. aeruginosa* metabolites as standards depending on the mass range of focus (*m/z* = 199.1698/529.3382/716.5231). Fast Fourier Transform filtering was applied to remove high frequency noise in the Fourier domain and the data peak-picked and mass recalibrated using the R package MALDIquant (31). The resulting data were saved in .csv format, ready for post-processing. Multivariate statistical analysis was performed on the data using the MetaboAnalyst 5.0 platform (32). Sum normalization, logarithmic transformation, and Pareto scaling were applied. Principal component analysis (PCA) and orthogonal projections to latent structures discriminant analysis (OPLS-DA) were used for data visualization and support vector machines (SVMs) for classification and feature selection. SVM models were validated using F1 to measure model accuracy, calculated from the model precision and recall.

### LC-MS/MS analysis

*P. aeruginosa* samples were prepared for LC-MS analysis by subculturing overnight cultures 1:50 into fresh LB medium, untreated or spiked with HOCl (3.2 mM). Once an OD_600_ of 1.0 was achieved, 100 µL of each culture was transferred to a microcentrifuge. Hank’s Balanced Salt Solution (100 µL, Gibco) was added and the mixture incubated at room temperature for 30 min. The mixture was then centrifuged at 3,270 × *g* for 5 min at 4°C, the supernatant removed, the remaining pellet washed using 1 mL ice-cold ammonium acetate solution (150 mM) in LC-MS grade H_2_O, centrifuged at 3,270 × *g* for 5 min at 4°C. The wash was repeated four more times. After the final supernatant removal, the pellet was stored at −80°C. LC-MS/MS analysis was performed by the National Phenome Centre (NPC) service using the lipidomics protocol in negative ion mode (33). Prior to analysis, the pellets were thawed and resuspended in ammonium acetate solution (150 mM). The final ratio of sample to IPA prior to injection was 1:4, as described in the protocol for human serum samples.

### Virulence factor assays

*P. aeruginosa* strains were grown overnight in 5 mL of LB broth in a 50-mL centrifuge tube (Falcon) and incubated overnight at 37°C at 180 rpm, and pyocyanin and elastase measured in the *P. aeruginosa* culture supernatants as previously described (34, 35).

## RESULTS

### Development of an LD-REIMS protocol to study the response of *P. aeruginosa* to oxidative stress

**Fig 1:**
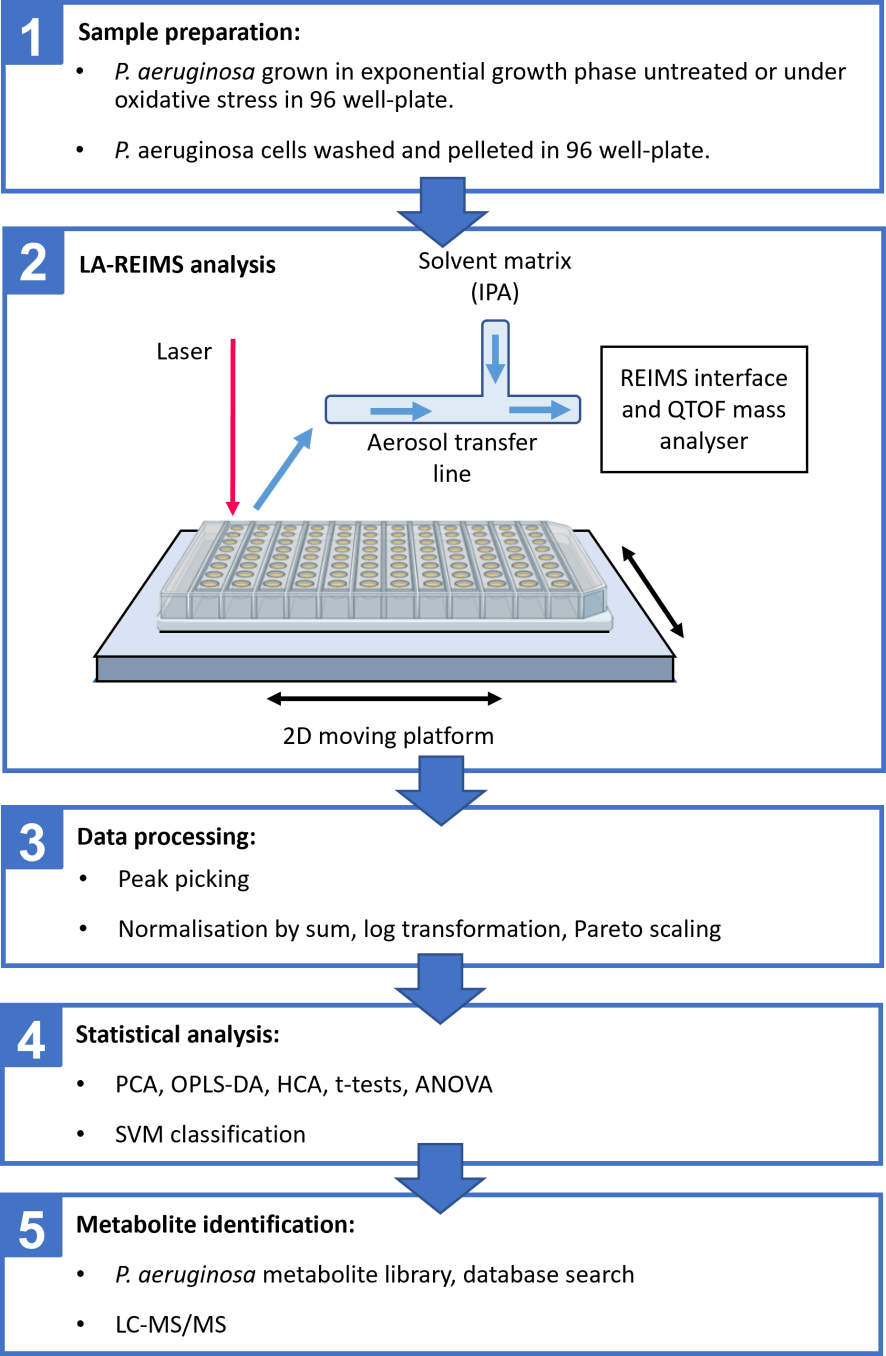
Protocol for high-throughput LD-REIMS analysis of *P. aeruginosa* exposed to oxidative stresses. (1) *P. aeruginosa* samples were grown in a 96-well plate, untreated or treated following balanced growth in exponential phase. This was achieved by inoculating 10 µL of *P. aeruginosa* stationary phase cultures into 200 µL of LB to give an initial OD_600_ of approximately 0.05, followed by growth for 4 h to an OD_600_ of approximately 0.8. The cultures were then pelleted, the pellets washed with ammonium acetate buffer and pelleted again. The time between starting to harvest the cultures and LD-REIMS analysis of the pellets was standardized to 60 ± 10 min. (2) Rapid heating and evaporation of the surface of each pellet was performed by moving the 96-well plate under a laser using a 2D moving platform. The resulting aerosol was transported to the LD-REIMS interface and analyzed using a QTOF mass analyzer. (3) Once the analysis was complete, the data were first processed using a peak-picking script. (4) Statistical analysis was performed on the processed data to determine which spectral features were significantly different between treated and untreated samples. Support vector machine (SVM) classification models were used to classify samples depending on treatment. (5) Metabolite identification was performed using a previous *P. aeruginosa* metabolite database generated in our lab and online databases. A selection of the most significantly impacted metabolites had their identity confirmed using LCMS/MS.

As REIMS has previously only been used to analyze bacterial colonies grown on agar, our first aim was to develop a protocol to analyze stressed, exponentially growing liquid cultures, with minimal disruption of cellular physiology between sampling and analysis, to investigate the metabolic changes occurring during the adaptive response of *P. aeruginosa* to oxidative stress. We grew *P. aeruginosa* cultures in a 96-well microtiter plate format with or without oxidative stress to exponential growth phase (OD_600_ ~0.8). The cells were then pelleted and washed with 150 mM ammonium acetate before transfer to the high-throughput LD-REIMS autosampler (Fig. 1).

**Fig 2:**
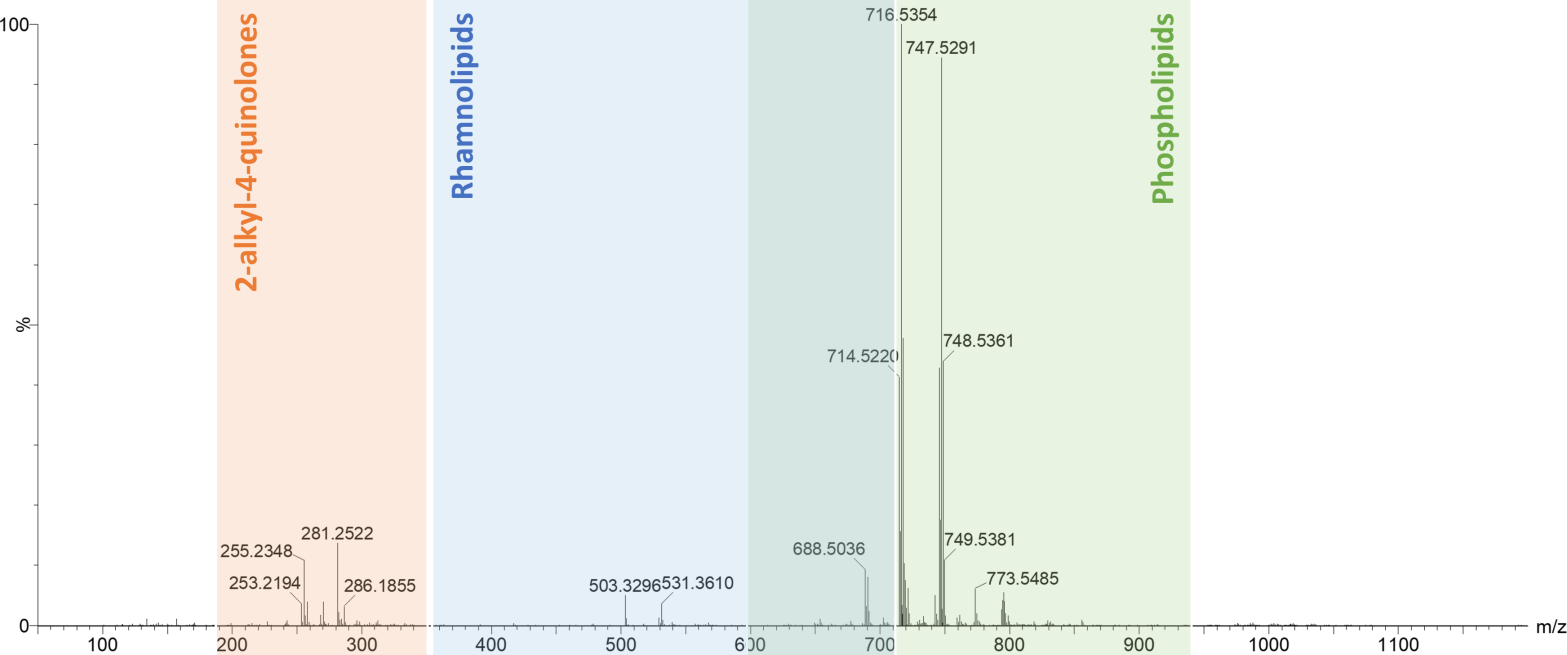
Typical mass spectrum of untreated *P. aeruginosa* obtained using the LD-REIMS protocol outlined in Fig. 1. The three main biomolecular regions are highlighted: 2-alkyl-4-quinolones (AHQs) at *m/z* 240–320, rhamnolipids at *m/z* 330–710, and phospholipids at *m/z* 600–900.

A typical LD-REIMS spectrum of *P. aeruginosa* samples prepared using this protocol (Fig. 2) showed a similar range of features to those described previously for analysis of *P. aeruginosa* colonies (20), with predominantly 2-alkyl-4-quinolones (AHQs) associated with the *Pseudomonas* quinolone signal (PQS) quorum sensing (QS) system detected in the range from *m/z* 240–320, rhamnolipids from *m/z* 330–710, and phospholipids from *m/z* 600 to 900.

### LD-REIMS analysis can distinguish between untreated and oxidatively stressed *P. aeruginosa* cultures

We then tested our LD-REIMS protocol for the detection of metabolic differences between untreated *P. aeruginosa* cultures and cultures stressed by growth in the presence of the oxidants HOCl, HOSCN, and H_2_O_2_, the superoxide generator MV and the reactive electrophilic compound MGO that has been shown to promote oxidative stress (36). One non-lethal concentration was chosen for each oxidant which led to a 1-h growth lag of the treated compared to the untreated cultures. This criterion was chosen to provide evidence that the bacteria were responding to the stress, adapting, and then resuming growth in its presence (Fig. S1). The selected concentrations were 3.2 mM HOCl, 0.35 mM HOSCN, 60 mM H_2_O_2_, 1.8 mM MGO, and 0.6 mM MV. Ten biological replicates were analyzed for each of the six sample classes (untreated, HOCl, HOSCN, H_2_O_2_, MGO, and MV).

**Fig 3:**
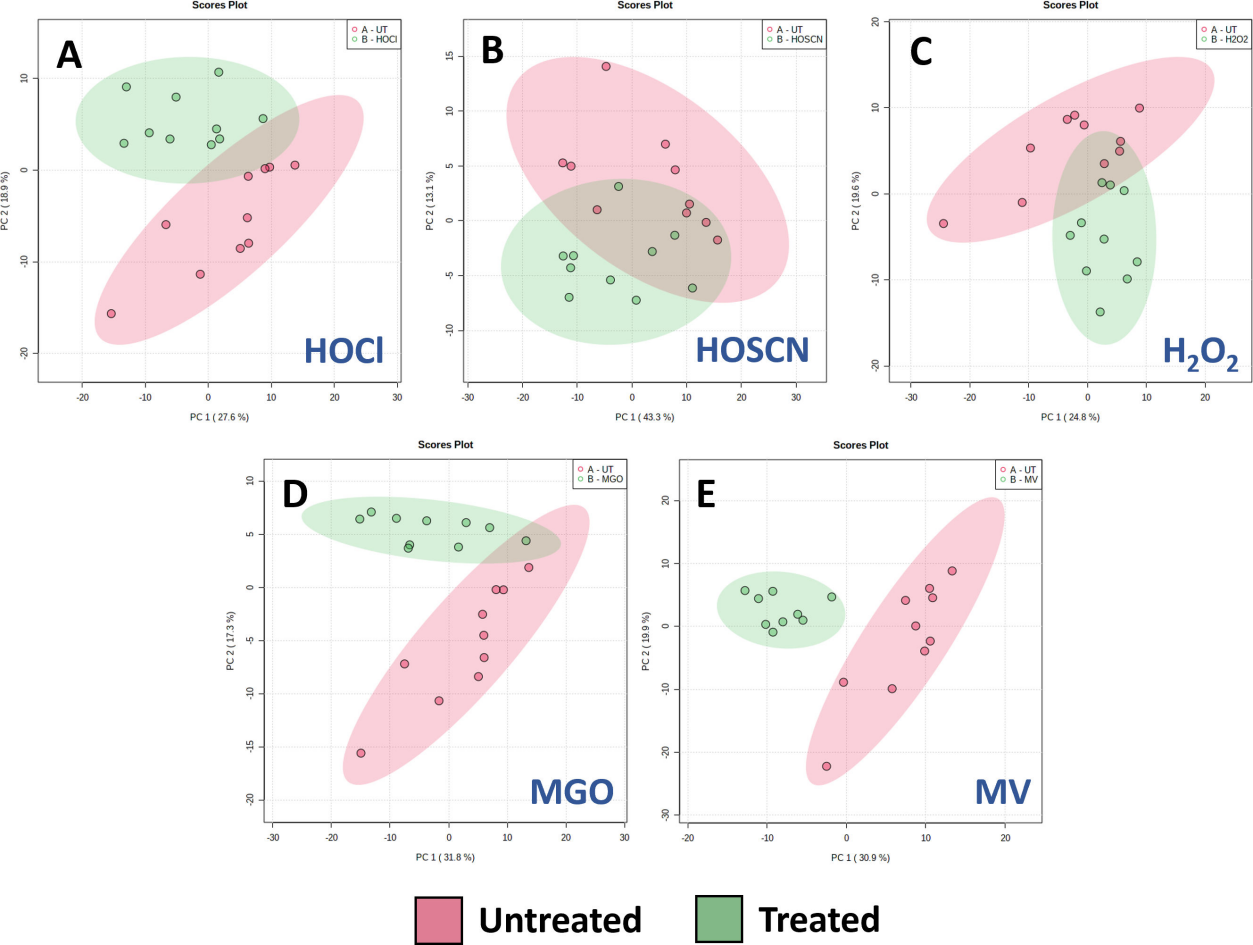
Statistical analysis of mass spectral data obtained by LD-REIMS by a two-group PCA of untreated and treated *P. aeruginosa* samples. (**A**) 3.2 mM HOCl, (**B**) 0.35 mM HOSCN, (**C**) 60 mM H_2_O_2_, (**D**) 1.8 mM MGO, and (**E**) 0.6 mM MV. Statistical analyses on samples (*n* = 10) were completed on the 50 to 1200 *m/z* range, after background subtraction and mass drift correction, using the MetaboAnalyst 5.0 platform. The plots all represent principal components 1 and 2. Shaded areas show 95% confidence intervals of the sample groups. Separation between untreated and treatment samples is clear for all oxidants.

Given the complexity of the LD-REIMS spectra across the different conditions, we first used PCA, a dimensionality reduction technique, to condense the data, allowing us to visualize high-level variance patterns in score plots (Fig. 3). Clear clustering of the untreated and treated classes can be observed for each oxidant, indicating that growth in their presence causes substantial metabolic divergence in the bacteria for the biomolecules detected by LD-REIMS.

**Fig 4:**
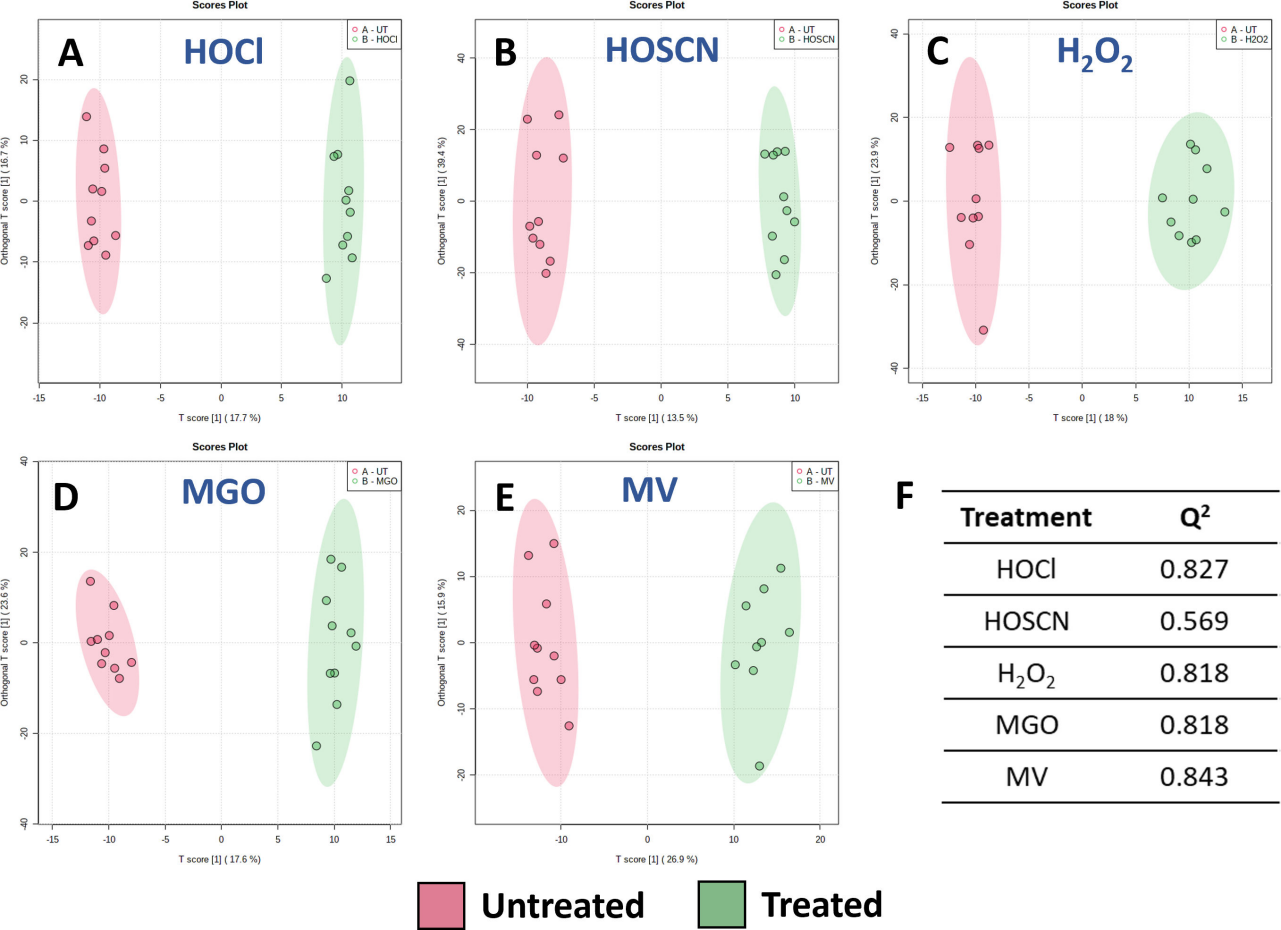
Statistical analysis of *P. aeruginosa* mass spectral data obtained by LD-REIMS by OPLS-DA analysis. Score plots generated based on OPLS-DA of untreated and treated *P. aeruginosa* samples (*n* = 10), (**A**) 3.2 mM HOCl, (**B**) 0.35 mM HOSCN, (**C**) 60 mM H_2_O_2_, (**D**) 1.8 mM MGO, and (**E**) 0.6 mM MV. Statistical analyses were completed on the 50 to 1200 *m/z* range, after background subtraction and mass drift correction, using the MetaboAnalyst 5.0 platform. (**F**) The model can separate the untreated and treated samples for each oxidant, cross-validated using the *Q*^2^ coefficient.

We then employed orthogonal projection to latent structures discriminant analysis (OPLS-DA) to confirm the discriminative power of LD-REIMS seen with PCA. OPLS-DA is a supervised modelling method, meaning the class labels are provided and the model identifies the variations in the data set that are most able to predict the given classes. While this approach provides greater power for class differentiation than PCA, it can be prone to overfitting, meaning the model fits the training data too well and cannot be generalized to new data sets. As one of the aims of this study was to identify biomarkers of oxidative stress that are universal to *P. aeruginosa* samples, it was particularly important to avoid overfitting. Therefore, we cross-validated the OPLS-DA models using the *Q*^2^ coefficient, a statistic that describes the predictive quality of the model, providing a measure of its reliability. Our analysis generated a *Q*^2^ value greater than 0.5 for each oxidant (Fig. 4), a commonly employed threshold for validation that suggests the model is less likely to be overfitted (37).

To provide further confirmation that the model was not overfitted, we tested the reproducibility of the data by performing an identical experiment on new cultures with the same level of biological replication (10 independent cultures) and then comparing the two independent data sets. OPLS-DA generated *Q*^2^ coefficients similar to the single-day analysis, indicating the discriminative power of the analysis was retained over different days, despite a reduction in separation in PCA scores plots for HOSCN, H_2_O_2_, and MGO that can be attributed to a batch effect (Figs S2 and S3). Overall, these analyses show that LD-REIMS can discriminate clearly and consistently between untreated and oxidatively stressed bacteria for a range of oxidants.

### Can LD-REIMS reliably classify *P. aeruginosa* based on stress exposure?

**Fig 5:**
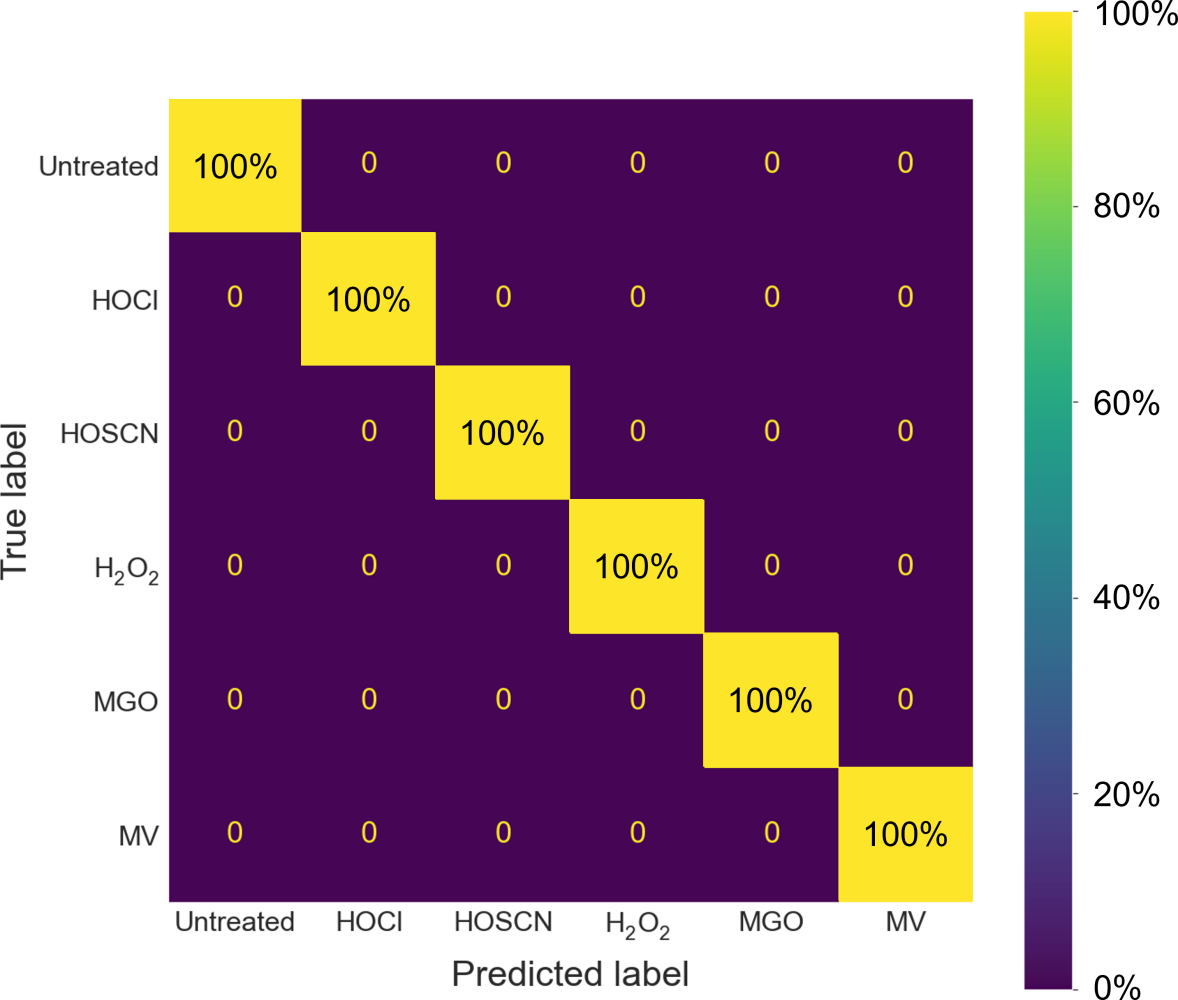
Confusion matrix for the SVM classification of *P. aeruginosa* samples exposed to oxidative stress conditions. The whole *m/z* ranges of the mass spectra acquired using LD-REIMS were employed to classify each sample into one of the six classes. The model was validated using *k*-fold cross-validation (*k* = 10). The samples were classified with 100% accuracy.

We next wanted to determine whether LD-REIMS can reliably classify samples based on the specific stress they have been exposed to. To achieve this, we first split the merged data set at random into a training set and a test set, in an 80:20 ratio. We then used the training set to build a support vector machine (SVM) for classification and validated this model using *k*-fold cross-validation (*k* = 10). SVMs are commonly employed for large metabolomics data sets due to their resistance to outliers and ability to handle noisy data (38). Using the model, the samples were classified depending on oxidant exposure with 100% accuracy (Fig. 5). The model was then tested using the test data set and the samples were again classified with 100% accuracy, demonstrating that LD-REIMS analysis provides sufficient discriminatory metabolic information to classify samples as treated with a specific oxidant with very high confidence.

### REIMS analysis can identify biomarkers of general oxidative stress and biomarkers specific to individual oxidants

We next investigated whether the metabolic changes detected in *P. aeruginosa* were specific to one or common to two or more oxidative stresses. Features were selected based on the relative importance of their linear regression coefficients obtained during SVM model training. In total, 333 spectral features were highlighted as significantly impacted by exposure to at least one oxidant. Of these, 54 (16.2%) were identified as unique metabolites (Table S2). Thirty-four of these were previously identified as *P. aeruginosa* metabolites using the same mass spectrometer (20). Twenty-four had their identity confirmed using LC-MS/MS (Table S3).

**Fig 6:**
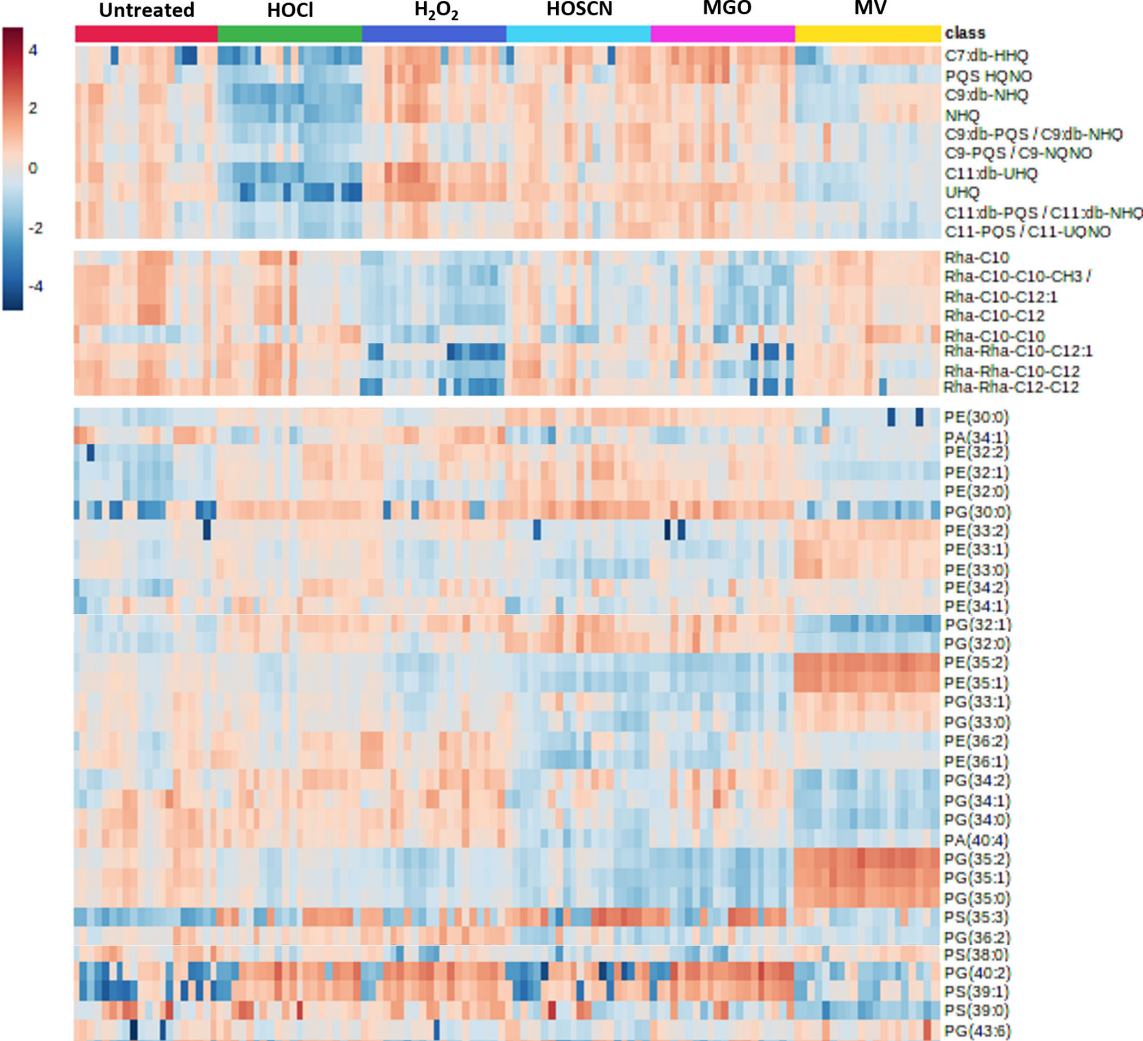
Heatmap visualisation of metabolite intensity changes in *P. aeruginosa* following treatment with oxidants. Color represents the variation in the normalized intensity of metabolite peaks in mass spectra, relative to the mean, where dark red means a higher intensity and blue a lower intensity. Metabolites are arranged into the three detected classes: PQS-associated AHQs, rhamnolipids, and phospholipids.

**Fig 7:**
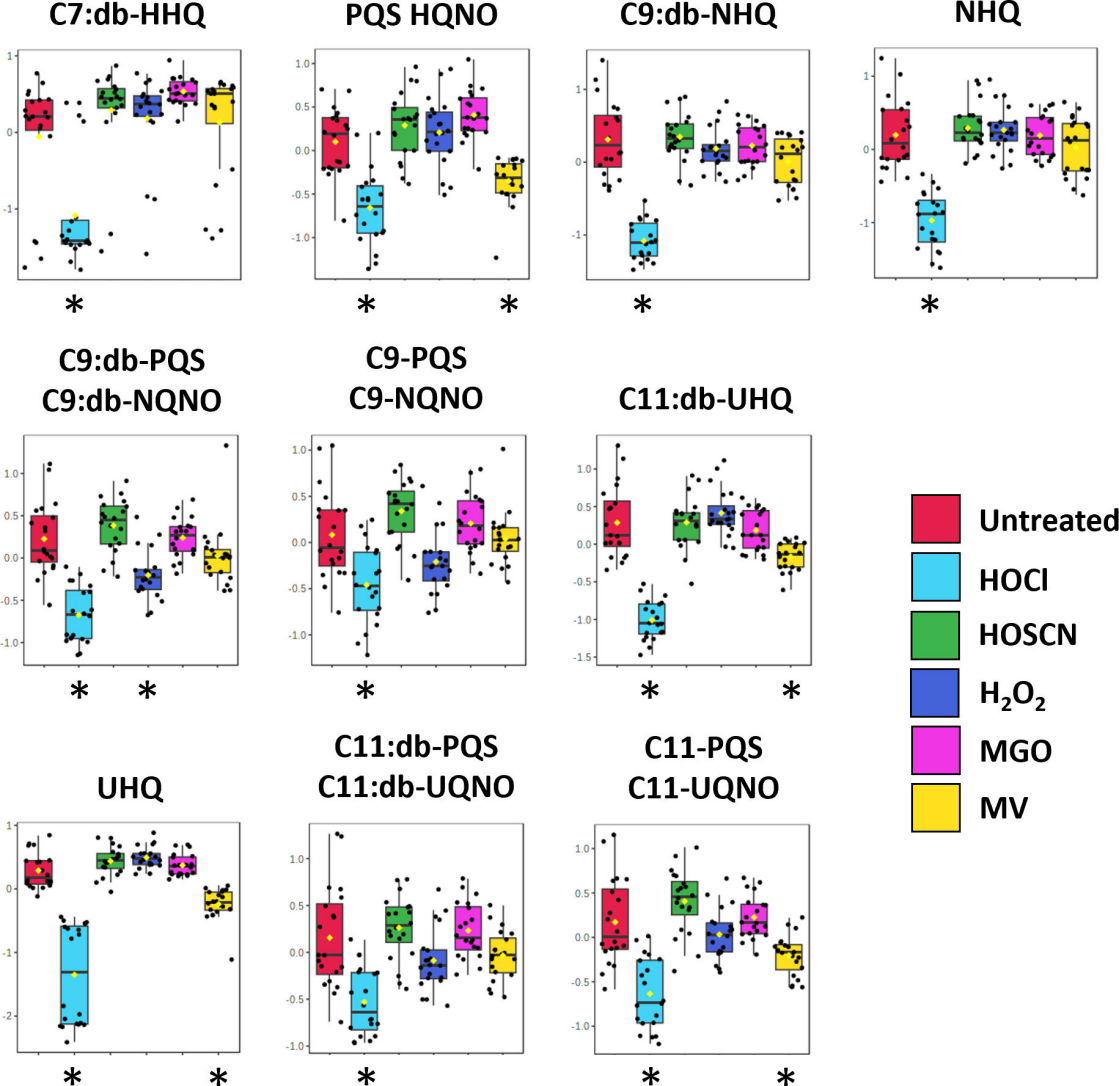
Changes in the intensities of PQS-associated quorum sensing molecules in *P. aeruginosa* exposed to oxidants. The box and whisker plots demonstrate the changes in AHQ intensities between different oxidative stresses. Exposure to HOCl caused a significant decrease in concentration of all 10 PQS-associated quorum sensing molecules identified using LD-REIMS, including the *Pseudomonas* quinolone signal (PQS) and its immediate precursor HHQ, the two most active signaling molecules for the PQS system. MV and H_2_O_2_ caused a decrease in the concentration of four AHQs and one AHQ, respectively. The threshold criterion for significance was a *P* value <0.05, determined using ANOVA and Fisher’s LSD post hoc analysis. Classes significantly different from untreated are highlighted with an asterisk. The box and whisker plots summarize the normalized intensity values (mean ± SD) for the metabolite.

**Fig 8:**
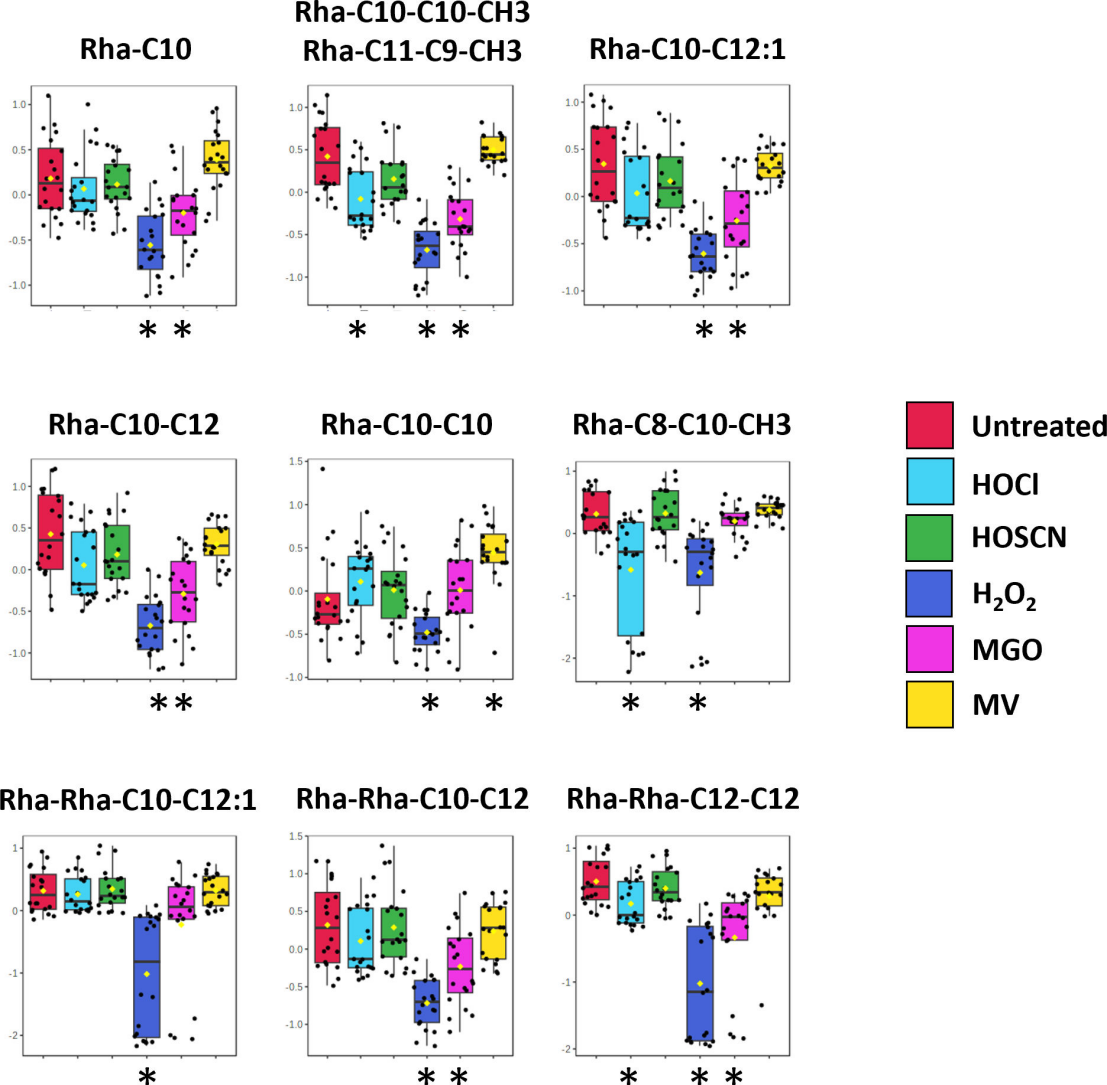
Changes in the intensities of rhamnolipids in *P. aeruginosa* exposed to oxidants. Exposure to H_2_O_2_ caused a decrease in concentration of all nine detected rhamnolipids in *P. aeruginosa*, while MGO, HOCl, and MV caused a decrease in concentration of five, three, and one rhamnolipid, respectively. The threshold criterion for significance was a *P* value <0.05, determined using ANOVA and Fisher’s LSD post hoc analysis. Classes significantly different from untreated are highlighted with an asterisk. The box and whisker plots summarize the normalized intensity values (mean ± SD) for the metabolite.

The changes in metabolite concentration observed in oxidatively stressed samples and the impact of each oxidant on the three classes of biomolecule are shown in Fig. 6 (see Table S4 for a list of the fold-changes in intensity and associated *P* values for each metabolite). These data allow us to draw conclusions about the specific metabolic impact of each oxidant. Exposure to HOCl significantly decreased the concentration of 10 AHQs (Fig. 6 and 7) to an extent not seen with any other oxidant, while also causing a decrease in the concentration of three rhamnolipids (Fig. 6 and 8). In contrast, 14 of the 21 phospholipids impacted by HOCl stress showed an increase in concentration, with the most notable exception being the phosphatidylglycerols PG(35:0), PG(35:1), and PG(35:2) which were significantly lower in concentration for all oxidants beside MV. No AHQ or rhamnolipid was impacted by exposure to HOSCN. However, 19 phospholipids were impacted by HOSCN stress. The phospholipids PE(30:0), PE(32:1), and PG(32:0) had higher concentrations in the HOSCN-treated samples than any other oxidant. Exposure to H_2_O_2_ had the strongest impact on rhamnolipid concentrations of all the oxidants (Fig. 8), while the impact on phospholipids was similar to the other treatments besides MV. The metabolite profiles of MGO-treated samples were broadly similar to those of H_2_O_2_-treated samples, having an almost identical phospholipid profile and having the second strongest impact on the detected rhamnolipids after H_2_O_2_. MV had a unique profile compared to the other treatments in the phospholipid region, causing an increase in concentration of several phospholipids that decreased in concentration for all other oxidants, and vice versa. The phospholipids PE(33:2), PE(33:1), and PE(30:0) increased in concentration only upon exposure to MV. MV also had a distinct impact on the growth of *P. aeruginosa* cultures, causing a reduction in the final OD_600_ reached in the stationary phase compared to untreated samples (Fig. S1). Each oxidant except HOSCN elicited at least one oxidant-specific metabolic change.

### Biomarkers of HOCl and HOSCN stress are conserved in *P. aeruginosa* clinical isolates

Our data have established that LD-REIMS can discriminate between oxidatively stressed and untreated and identified biomarkers specific to exposure to different oxidants in PA14 samples, a commonly used lab strain of *P. aeruginosa*. For these findings to have potential use in the investigation of *P. aeruginosa* infections *in vivo*, for example, to establish whether bacteria are exposed to these stresses during infection, we needed to demonstrate that these specific metabolomic responses are conserved in clinical strains. Therefore, we carried out LD-REIMS analysis on 10 *P*. *aeruginosa* strains isolated from cystic fibrosis (CF) lung infections and compared their untreated and HOCl-treated metabolomes using LD-REIMS.

Separation in PCA scores plots between treated and untreated samples was reduced compared to the lab strain analysis (Fig. S4 and S5). However, this included the PA14 control, suggesting the reduced discriminative power was due to the lower number of replicates (*n* = 6 compared to *n* = 20).

We then tracked in the clinical isolates the biomarkers of HOCl and HOSCN established using PA14. The decrease in concentration of AHQs was conserved in the HOCl-treated clinical isolates (Table S5). C9:db-NHQ, C11:db-UHQ, and UHQ were conserved in all or all but one isolate, suggesting any impact of HOCl on the PQS system is not limited to the laboratory strain. The rhamnolipids and phospholipid biomarkers were poorly conserved in both the clinical isolates and the PA14 control. The HOSCN biomarkers were very well conserved (Table S6). All biomarkers besides PA(34:1) and PS(38:0) were conserved in the majority of clinical isolates. Only C13 and C25 presented with fewer than 50% of the biomarkers.

### HOCl-stress reduces quorum sensing-mediated virulence factor levels

**Fig 9:**
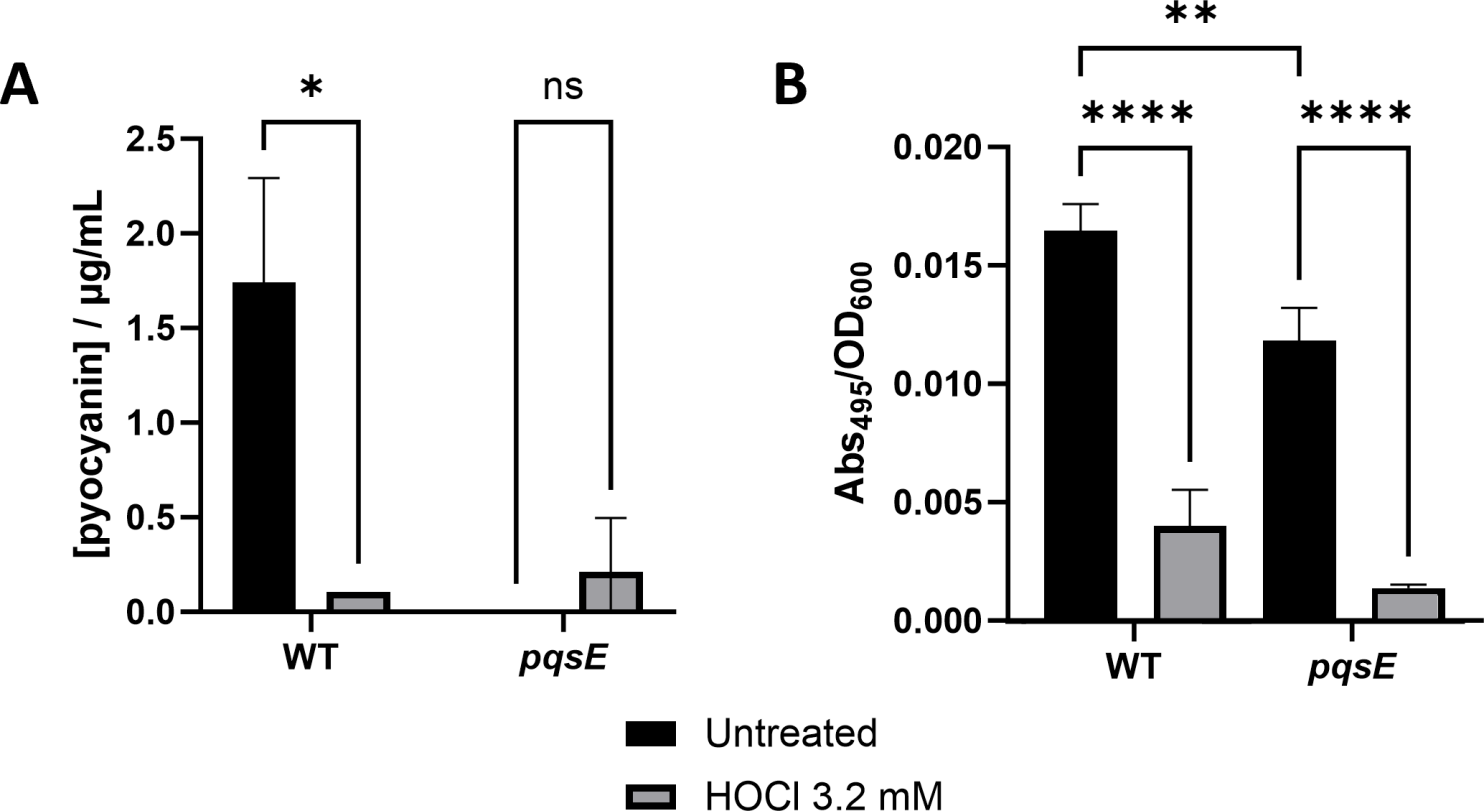
HOCl stress reduces the levels of the virulence factors pyocyanin and elastase in *P. aeruginosa*. *P. aeruginosa* wild-type cultures and a *pqsE* mutant, that is defective in a gene essential for PQS synthesis, were grown in the presence and absence of 3.2 mM HOCl, and their pyocyanin (**A**) and elastase (**B**) levels were analyzed (*n* = 3). Statistical analysis was performed using one-way analysis of variance with Tukey’s multiple-comparison post hoc test. *, *P* < 0.05; **, *P* < 0.01; ****, *P* < 0.0001.

The decrease in concentration of 10 AHQs associated with the PQS quorum sensing system on exposure to HOCl was one of the strongest patterns observed in our data. While it is not possible to say whether the decrease in concentration is due to a reduction in their production or to direct oxidation of the metabolites, the outcome is likely to be a decrease in the efficacy of the molecules to modulate the PQS-mediated quorum sensing response of *P. aeruginosa*. To investigate this, we measured the concentration of pyocyanin and elastase, virulence factors known to be regulated by the PQS system, in wild-type cultures grown in the presence of HOCl. We compared the concentrations to those in *pqsE* mutants, which are mutated in genes required for PQS synthesis and are expected to produce little or no pyocyanin and elastase (39). Pyocyanin and elastase were clearly made by the wild-type strain but when grown in the presence of HOCl the levels were significantly affected (Fig. 9), with pyocyanin not detectable at concentrations above those in the *pqsE* mutant and elastase present at less than 25% of the concentration seen in the untreated bacteria. To test whether the decrease in pyocyanin was due to a decrease in production or direct oxidation, we treated stationary phase cultures in which pyocyanin was already present with HOCl and measured the concentration over time. We did not see a difference in the concentration of pyocyanin in treated cultures (Fig. S8), suggesting that HOCl does not cause direct oxidation of pyocyanin.

## DISCUSSION

### LD-REIMS can be used to confidently distinguish between oxidatively stressed *P. aeruginosa* cultures

In this study, we tested LD-REIMS as a novel technique for the metabolic profiling of *P. aeruginosa*, with the aim to identify biomarkers of oxidative stress and to provide insight into the oxidative stress protection systems employed by the bacteria. REIMS has been employed for a range of microbiological studies, most frequently for bacterial identification and classification (22, 40–43), as well as metabolic screening of yeast colonies (21) and imaging (44). A study in the Takats’ lab successfully classified *P. aeruginosa* isolates to a strain level with 81% accuracy and identified a number of *P. aeruginosa* metabolites (20).

While REIMS has been established for the analysis of bacterial colonies, the LD-REIMS protocol reported here allows for the analysis of pelleted samples, meaning bacterial samples grown in liquid, including high salt media that REIMS would be intolerant toward, can be analyzed in a high-throughput manner. We analyzed and compared the metabolomes of *P. aeruginosa* grown in the absence and presence of oxidative stresses, with the only sample preparation requirement being the centrifugation and washing of the samples just prior to analysis. Using our approach, 96 samples can be analyzed in 20 min, and samples are analyzed within 60 min of the start of sample preparation. Additionally, this was the first reported application of a 3 µm laser for high-throughput REIMS, after ongoing research found that a 3 µm, shorter pulse width laser led to increased ion yields compared to the original CO_2_ setup (30). The limitation of REIMS is that it has a restricted metabolic coverage, largely detecting hydrophobic molecules in the surface layers of bacteria, but molecules that will be first to come into contact with an externally applied stress.

We used an SVM model to classify *P. aeruginosa* PA14 samples based on the specific oxidative stress they were grown in with 100% accuracy. This result was reproducible, with classification accuracy consistent for samples prepared and analyzed on different days.

These experiments were performed on the lab strain PA14, a burn wound isolate that represents the most common clonal group worldwide and is more virulent than other commonly used strains, PAO1 and PAK (45)(45). It is now recognized that it is important not to rely on analysis of a limited number of laboratory reference strains but to extend to natural isolates, including clinical strains (46). For this reason, we extended our LD-REIMS study to clinical isolates from CF patients. The biomarkers of HOCl and HOSCN stress were conserved in the clinical isolates. While the small number of clinical strains used in this study do not provide sufficient statistical power to look for conserved lifestyle specific biomarkers, the speed and simplicity of the LD-REIMS approach make this a feasible future research objective.

### A unique metabolic profile was recorded for each oxidant

The observed classification accuracy of 100% indicates that growth in the presence of different oxidants impacts the *P. aeruginosa* metabolome in a manner unique to each oxidant, at least for the metabolites detectable using LD-REIMS. We were able to use a combination of univariate and multivariate analyses to find the metabolites with significantly different intensities between sample classes. These included representatives from all three of the main classes of *P. aeruginosa* metabolites detected using LD-REIMS: AHQs associated with the PQS quorum sensing system, rhamnolipids, and phospholipids (20).

Of the oxidants, HOCl and MV (superoxide) had the greatest impact on the *P. aeruginosa* metabolome, with both seen to cluster away from the untreated samples using PLS-DA. HOSCN, H_2_O_2_, and MGO did not separate from untreated when all oxidants were analyzed together; however, clear separation from untreated samples was observed when analyzed independently using two-group PCA and OPLS-DA, and all samples were classified with 100% accuracy. This suggests HOCl and MV have a broader impact on the metabolome, disrupting the concentrations of a greater range of molecules, while the other three impact fewer metabolites but still leave a consistent and unique mark on the metabolome. The greater impact of HOCl matches with the reaction profiles of the oxidants. HOCl is a stronger oxidant than H_2_O_2_ or HOSCN, and able to react with a wide range of bacterial substrates including thiols and amines (47). HOSCN reacts almost solely with thiol groups, while H_2_O_2_ only reacts directly with thiols and transition metal centres, due to the high activation energy of its reactions (48). Reaction profiles are not sufficient to explain the greater impact of MV, as superoxide has a low reactivity in aqueous solutions (1). However, the use of methyl viologen as an intermediary for superoxide generation may explain the impact on the metabolome. MV (also known as paraquat) is non-polar and can therefore pass through bacterial membranes, where superoxide is generated within the cell. The intracellular production of MV would likely have a significantly different impact on the metabolome compared to the other oxidants, which may cause more damage at the cell membrane. This is particularly relevant for this study, as the detected molecules are membrane-associated or extracellular.

### HOCl has a specific impact on AHQs associated with the PQS quorum sensing system

Our most striking finding was the decrease in concentration of 10 AHQs associated with the PQS quorum sensing system after exposure to HOCl. Among these are 2-heptyl-3-hydroxy-4(1H)-quinolone, also known as the PQS, the most active signal molecule of the PQS system that regulates the production of virulence factors including elastase, rhamnolipids, pyocyanin, and siderophores, such as pyoverdine, alongside inducing its own production (49). The decrease in concentration of AHQs was also seen in the clinical isolates (Table S6), clearly showing that the impact of HOCl on the PQS system is replicated in clinical strains.

PQS and other AHQs are released extracellularly by *P. aeruginosa* where their concentration acts as a proxy of the local bacterial population and subsequently dictates the expression of PQS-dependent genes. Due to their alkyl chain moiety, AHQs are hydrophobic and rely on membrane vesicles (MVs) for transport to and excretion from the cell. Their association with MVs and the cell envelope explains their prevalence in LD-REIMS *P. aeruginosa* mass spectra alongside phospholipids and rhamnolipids. The PQS system is associated with the oxidative stress response of *P. aeruginosa*, having been shown to act as both an antioxidant and pro-oxidant in different contexts (49–51). Its antioxidant effect is attributed to its nature as a strong electron donor due to its bisoxygenated aromatic structure, able to reduce intracellular reactive oxygen species (ROS) levels through direct reaction with oxidants. However, it has also been shown to sensitize *P. aeruginosa* to killing by H_2_O_2_ and oxidative stress-mediated via ultraviolet radiation (50, 52). This pro-oxidant effect is thought to be due to the ability of PQS to chelate Fe^3+^ ions, resulting in the formation of free radicals.

It is unclear whether the decrease in concentration of the AHQs is due to HOCl inhibiting their production or to their direct oxidation. However, irrespective of the mechanism, the phenotypic impact is clear as the PQS-regulated virulence factor pyocyanin was undetectable and elastase concentration was reduced by >75% in HOCl stressed cultures. In addition, we showed that this was not due to oxidation of pyocyanin by HOCl, supporting a model whereby HOCl prevents the production of AHQs. However, we demonstrated previously that the transcriptional regulator RclR, which is required for protection of *P. aeruginosa* against HOCl stress, *positively* regulates the expression of the *phz* genes that encode the pyocyanin biosynthesis pathway in the presence of HOCl (7). While the pyocyanin phenotype is clear, these data indicate complexity in the effect of HOCl on pyocyanin formation and the associated regulatory circuits that need further investigation. An additional consideration is that the decrease in concentration of the AHQs in the presence of HOCl would additionally remove the potential antioxidant properties of the PQS system. Multiple lines of evidence support the importance of PQS-mediated quorum sensing and its upregulation of virulence factor production in the pathogenicity of *P. aeruginosa* (39, 53, 54,4, 41, 54). Therefore, our findings lead us to hypothesize that, during infection, neutrophil-generated HOCl formation results in the inhibition of virulence factor formation by *P. aeruginosa* due to its effect on PQS-mediated quorum sensing, and that this augments the impact of direct neutrophil-killing of bacteria by reducing their pathogenicity.

## Data Availability

The data described in this article are openly available on the MassIVE data repository at ftp://MSV000093056@massive.ucsd.edu.

## SUPPLEMENTAL MATERIAL

Supplemental figures and tables msystems.01165-23-s0001.docx Figures S1 to S8 and Tables S1 to S6.

ASM does not own the copyrights to Supplemental Material that may be linked to, or accessed through, an article. The authors have granted ASM a non-exclusive, world-wide license to publish the Supplemental Material files. Please contact the corresponding author directly for reuse.

## References

[1] Halliwell B, Gutteridge JMC. 2015. Free radicals in biology and medicine. Oxford University Press.

[2] Marciano BE, Spalding C, Fitzgerald A, Mann D, Brown T, Osgood S, Yockey L, Darnell DN, Barnhart L, Daub J, et al.. 2015. Common severe infections in chronic granulomatous disease. Clin Infect Dis 60:1176–1183. doi:10.1093/cid/ciu1154 25537876 PMC4400412

[3] Lam PL, Wong RSM, Lam KH, Hung LK, Wong MM, Yung LH, Ho YW, Wong WY, Hau DKP, Gambari R, Chui CH. 2020. The role of reactive oxygen species in the biological activity of antimicrobial agents: an updated mini review. Chem Biol Interact 320:109023. doi:10.1016/j.cbi.2020.109023 32097615

[4] Palma M, DeLuca D, Worgall S, Quadri LEN. 2004. Transcriptome analysis of the response of Pseudomonas aeruginosa to hydrogen peroxide. J Bacteriol 186:248–252. doi:10.1128/JB.186.1.248-252.2004 14679246 PMC303446

[5] Rada B, Leto TL. 2008. Oxidative innate immune defenses by NOx/Duox family NADPH oxidases. Contrib Microbiol 15:164–187. doi:10.1159/000136357 18511861 PMC2776633

[6] Klebanoff SJ, Kettle AJ, Rosen H, Winterbourn CC, Nauseef WM. 2013. Myeloperoxidase: a front-line defender against phagocytosed microorganisms. J Leukoc Biol 93:185–198. doi:10.1189/jlb.0712349 23066164 PMC3545676

[7] Farrant KV, Spiga L, Davies JC, Williams HD. 2020. Response of Pseudomonas aeruginosa to the innate immune system-derived oxidants hypochlorous acid and hypothiocyanous acid. J Bacteriol 203:300–320. doi:10.1128/JB.00300-20 PMC7950407 33106346

[8] Nontaleerak B, Eurtivong C, Weeraphan C, Buncherd H, Chokchaichamnankit D, Srisomsap C, Svasti J, Sukchawalit R, Mongkolsuk S. 2023. The redox-sensing mechanism of Agrobacterium tumefaciens NieR as a thiol-based oxidation sensor for hypochlorite stress. Free Radic Biol Med 208:211–220. doi:10.1016/j.freeradbiomed.2023.08.002 37544488

[9] Groitl B, Dahl JU, Schroeder JW, Jakob U. 2017. Pseudomonas aeruginosa defense systems against microbicidal oxidants. Mol Microbiol 106:335–350. doi:10.1111/mmi.13768 28795780 PMC5653425

[10] Schäfer K-C, Dénes J, Albrecht K, Szaniszló T, Balog J, Skoumal R, Katona M, Tóth M, Balogh L, Takáts Z. 2009. In vivo, in situ tissue analysis using rapid evaporative ionization mass spectrometry. Angew Chem Int Ed Engl 48:8240–8242. doi:10.1002/anie.200902546 19746375

[11] Feider CL, Krieger A, DeHoog RJ, Eberlin LS. 2019. Ambient ionization mass spectrometry: recent developments and applications. Anal Chem 91:4266–4290. doi:10.1021/acs.analchem.9b00807 30790515 PMC7444024

[12] Ellis BM, Fischer CN, Martin LB, Bachmann BO, McLean JA. 2019. Spatiochemically profiling microbial interactions with membrane scaffolded desorption electrospray Ionization-ion mobility-imaging mass spectrometry and unsupervised segmentation. Anal Chem 91:13703–13711. doi:10.1021/acs.analchem.9b02992 31600444 PMC7331455

[13] Parrot D, Papazian S, Foil D, Tasdemir D. 2018. Imaging the unimaginable: desorption electrospray ionization - imaging mass spectrometry (DESI-IMS) in natural product research. Planta Med 84:584–593. doi:10.1055/s-0044-100188 29388184 PMC6053038

[14] Rath CM, Alexandrov T, Higginbottom SK, Song J, Milla ME, Fischbach MA, Sonnenburg JL, Dorrestein PC. 2012. Molecular analysis of model gut microbiotas by imaging mass spectrometry and nanodesorption electrospray ionization reveals dietary metabolite transformations. Anal Chem 84:9259–9267. doi:10.1021/ac302039u 23009651 PMC3711173

[15] Song Y, Talaty N, Datsenko K, Wanner BL, Cooks RG. 2009. In vivo recognition of Bacillus subtilis by desorption electrospray ionization mass spectrometry (DESI-MS). Analyst 134:838–841. doi:10.1039/b900069k 19381372

[16] Zhang JI, Talaty N, Costa AB, Xia Y, Tao WA, Bell R, Callahan JH, Cooks RG. 2011. Rapid direct lipid profiling of bacteria using desorption electrospray ionization mass spectrometry. Int J Mass Spectrom 301:37–44. doi:10.1016/j.ijms.2010.06.014

[17] Hamid AM, Jarmusch AK, Pirro V, Pincus DH, Clay BG, Gervasi G, Cooks RG. 2014. Rapid discrimination of bacteria by paper spray mass spectrometry. Anal Chem 86:7500–7507. doi:10.1021/ac501254b 25014713

[18] Parsiegla G, Shrestha B, Carrière F, Vertes A. 2012. Direct analysis of phycobilisomal antenna proteins and metabolites in small cyanobacterial populations by laser ablation electrospray ionization mass spectrometry. Anal Chem 84:34–38. doi:10.1021/ac202831w 22141353

[19] Pierce CY, Barr JR, Cody RB, Massung RF, Woolfitt AR, Moura H, Thompson HA, Fernandez FM. 2007. Ambient generation of fatty acid methyl ester ions from bacterial whole cells by direct analysis in real time (DART) mass spectrometry. Chem Commun (Camb): 807–809. doi:10.1039/b613200f 17308638

[20] Bardin EE, Cameron SJS, Perdones-Montero A, Hardiman K, Bolt F, Alton EWFW, Bush A, Davies JC, Takáts Z. 2018. Metabolic phenotyping and strain characterisation of Pseudomonas aeruginosa isolates from cystic fibrosis patients using rapid evaporative ionisation mass spectrometry. Sci Rep 8:10952. doi:10.1038/s41598-018-28665-7 30026575 PMC6053451

[21] Gowers GOF, Cameron SJS, Perdones-Montero A, Bell D, Chee SM, Kern M, Tew D, Ellis T, Takáts Z. 2019. Off-colony screening of biosynthetic libraries by rapid laser-enabled mass spectrometry. ACS Synth Biol 8:2566–2575. doi:10.1021/acssynbio.9b00243 31622554

[22] Cameron SJS, Perdones-Montero A, Van Meulebroek L, Burke A, Alexander-Hardiman K, Simon D, Schaffer R, Balog J, Karancsi T, Rickards T, Rebec M, Stead S, Vanhaecke L, Takáts Z. 2021. Sample preparation free mass spectrometry using laser-assisted rapid evaporative ionization mass spectrometry: applications to microbiology, metabolic biofluid phenotyping, and food authenticity. J Am Soc Mass Spectrom 32:1393–1401. doi:10.1021/jasms.0c00452 33980015

[23] Van Meulebroek L, Cameron S, Plekhova V, De Spiegeleer M, Wijnant K, Michels N, De Henauw S, Lapauw B, Takats Z, Vanhaecke L. 2020. Rapid LA-REIMS and comprehensive UHPLC-HRMS for metabolic phenotyping of feces. Talanta 217:121043. doi:10.1016/j.talanta.2020.121043 32498888

[24] Plekhova V, Van Meulebroek L, De Graeve M, Perdones-Montero A, De Spiegeleer M, De Paepe E, Van de Walle E, Takats Z, Cameron SJS, Vanhaecke L. 2021. Rapid ex vivo molecular fingerprinting of biofluids using laser-assisted rapid evaporative ionization mass spectrometry. Nat Protoc 16:4327–4354. doi:10.1038/s41596-021-00580-8 34341579

[25] Wijnant K, Van Meulebroek L, Pomian B, De Windt K, De Henauw S, Michels N, Vanhaecke L. 2020. Validated ultra-high-performance liquid chromatography hybrid high-resolution mass spectrometry and laser-assisted rapid evaporative ionization mass spectrometry for salivary metabolomics. Anal Chem 92:5116–5124. doi:10.1021/acs.analchem.9b05598 32150679

[26] Liberati NT, Urbach JM, Miyata S, Lee DG, Drenkard E, Wu G, Villanueva J, Wei T, Ausubel FM. 2006. An ordered, nonredundant library of Pseudomonas aeruginosa strain PA14 transposon insertion mutants. Proc Natl Acad Sci U S A 103:2833–2838. doi:10.1073/pnas.0511100103 16477005 PMC1413827

[27] Chandler JD, Nichols DP, Nick JA, Hondal RJ, Day BJ. 2013. Selective metabolism of hypothiocyanous acid by mammalian thioredoxin reductase promotes lung innate immunity and antioxidant defense. J Biol Chem 288:18421–18428. doi:10.1074/jbc.M113.468090 23629660 PMC3689984

[28] Nagy P, Jameson GNL, Winterbourn CC. 2009. Kinetics and mechanisms of the reaction of hypothiocyanous acid with 5-thio-2-nitrobenzoic acid and reduced glutathione. Chem Res Toxicol 22:1833–1840. doi:10.1021/tx900249d 19821602

[29] Simon D, Horkovics-Kovats GS, Xiang Y, Abda J, Papanastasiou D, Ho H-Y, Wang H, Schäffer R, Karancsi T, Mroz A, Lagache L, Balog J, Fournier I, Bunch J, Takats Z. 2023. Sample preparation free tissue imaging using laser desorption – rapid evaporative ionisation mass spectrometry (LD-REIMS). ChemRxiv. doi:10.26434/chemrxiv-2023-p2g9h-v2

[30] Jones EA, Simon D, Karancsi T, Balog J, Pringle SD, Takats Z. 2019. Matrix assisted rapid evaporative ionization mass spectrometry. Anal Chem 91:9784–9791. doi:10.1021/acs.analchem.9b01441 31194519

[31] Gibb S, Strimmer K. 2012. MALDIquant: a versatile R package for the analysis of mass spectrometry data. Bioinformatics 28:2270–2271. doi:10.1093/bioinformatics/bts447 22796955

[32] Pang Z, Chong J, Zhou G, Anderson de Lima Morais D, Chang L, Barrette M, Gauthier C, Jacques PE, Li S, Xia J. 2021. Metaboanalyst 5.0: Narrowing the gap between raw spectra and functional insights. Nucleic Acids Res 49:W388–W396. doi:10.1093/nar/gkab382 34019663 PMC8265181

[33] Lewis M, Chekmeneva E, Camuzeaux S, Sands C, Yuen A, David M, Salam A, Chappell K, Cooper B, Haggart G, Maslen L, Gómez-Romero M, Horneffer-van der Sluis V, Correia G, Takats Z. 2022. An open platform for large scale LC-MS-based metabolomics. ChemRxiv. doi:10.26434/chemrxiv-2022-nq9k0

[34] Essar DW, Eberly L, Hadero A, Crawford IP. 1990. Identification and characterization of genes for a second anthranilate synthase in Pseudomonas aeruginosa: interchangeability of the two anthranilate synthase and evolutionary implications. J Bacteriol 172:884–900. doi:10.1128/jb.172.2.884-900.1990 2153661 PMC208517

[35] Filloux A, Ramos JL. 2014. Pseudomonas methods and protocols. Vol. 1149. 10.1007/978-1-4939-0473-0 24936603

[36] Figarola JL, Singhal J, Rahbar S, Awasthi S, Singhal SS. 2014. LR-90 prevents methylglyoxal-induced oxidative stress and apoptosis in human endothelial cells. Apoptosis 19:776–788. doi:10.1007/s10495-014-0974-3 24615331 PMC4169289

[37] Triba MN, Le Moyec L, Amathieu R, Goossens C, Bouchemal N, Nahon P, Rutledge DN, Savarin P. 2015. PLS/OPLS models in metabolomics: the impact of permutation of dataset rows on the K-fold cross-validation quality parameters. Mol Biosyst 11:13–19. doi:10.1039/c4mb00414k 25382277

[38] Mahadevan S, Shah SL, Marrie TJ, Slupsky CM. 2008. Analysis of metabolomic data using support vector machines. Anal Chem 80:7562–7570. doi:10.1021/ac800954c 18767870

[39] Bala A, Kumar L, Chhibber S, Harjai K. 2015. Augmentation of virulence related traits of pqs mutants by Pseudomonas quinolone signal through membrane vesicles. J Basic Microbiol 55:566–578. doi:10.1002/jobm.201400377 25283438

[40] Strittmatter N, Rebec M, Jones EA, Golf O, Abdolrasouli A, Balog J, Behrends V, Veselkov KA, Takats Z. 2014. Characterization and identification of clinically relevant microorganisms using rapid evaporative ionization mass spectrometry. Anal Chem 86:6555–6562. doi:10.1021/ac501075f 24896667

[41] Bolt F, Cameron SJS, Karancsi T, Simon D, Schaffer R, Rickards T, Hardiman K, Burke A, Bodai Z, Perdones-Montero A, Rebec M, Balog J, Takats Z. 2016. Automated high-throughput identification and characterization of clinically important bacteria and fungi using rapid evaporative ionization mass spectrometry. Anal Chem 88:9419–9426. doi:10.1021/acs.analchem.6b01016 27560299

[42] Bodai Z, Cameron S, Bolt F, Simon D, Schaffer R, Karancsi T, Balog J, Rickards T, Burke A, Hardiman K, Abda J, Rebec M, Takats Z. 2018. Effect of electrode geometry on the classification performance of rapid evaporative ionization mass spectrometric (REIMS) bacterial identification. J Am Soc Mass Spectrom 29:26–33. doi:10.1007/s13361-017-1818-5 29038998 PMC5785610

[43] Cameron SJS, Bolt F, Perdones-Montero A, Rickards T, Hardiman K, Abdolrasouli A, Burke A, Bodai Z, Karancsi T, Simon D, Schaffer R, Rebec M, Balog J, Takáts Z. 2016. Rapid evaporative ionisation mass spectrometry (REIMS) provides accurate direct from culture species identification within the genus Candida. Sci Rep 6:36788. doi:10.1038/srep36788 27841356 PMC5107957

[44] Golf O, Strittmatter N, Karancsi T, Pringle SD, Speller AVM, Mroz A, Kinross JM, Abbassi-Ghadi N, Jones EA, Takats Z. 2015. Rapid evaporative ionization mass spectrometry imaging platform for direct mapping from bulk tissue and bacterial growth media. Anal Chem 87:2527–2534. doi:10.1021/ac5046752 25671656

[45] Grace A, Sahu R, Owen DR, Dennis VA. 2022. Pseudomonas aeruginosa reference strains PAO1 and PA14: a genomic, phenotypic, and therapeutic review. Front Microbiol 13:1023523. doi:10.3389/fmicb.2022.1023523 36312971 PMC9607943

[46] Hefnawy A, Cantizani J, Peña I, Manzano P, Rijal S, Dujardin J-C, De Muylder G, Martin J. 2018. Importance of secondary screening with clinical isolates for anti-leishmania drug discovery. Sci Rep 8:11765. doi:10.1038/s41598-018-30040-5 30082744 PMC6078976

[47] Winterbourn CC, Kettle AJ, Hampton MB. 2016. Reactive oxygen species and neutrophil function. Annu Rev Biochem 85:765–792. doi:10.1146/annurev-biochem-060815-014442 27050287

[48] Winterbourn CC, Hampton MB. 2008. Thiol chemistry and specificity in redox signaling. Free Radic Biol Med 45:549–561. doi:10.1016/j.freeradbiomed.2008.05.004 18544350

[49] García-Reyes S, Soberón-Chávez G, Cocotl-Yanez M. 2020. The third quorum-sensing system of Pseudomonas aeruginosa: Pseudomonas quinolone signal and the enigmatic PqsE protein. J Med Microbiol 69:25–34. doi:10.1099/jmm.0.001116 31794380

[50] Häussler S, Becker T. 2008. The Pseudomonas quinolone signal (PQS) balances life and death in Pseudomonas aeruginosa populations. PLoS Pathog 4:e1000166. doi:10.1371/journal.ppat.1000166 18818733 PMC2533401

[51] Bollinger N, Hassett DJ, Iglewski BH, Costerton JW, McDermott TR. 2001. Gene expression in Pseudomonas aeruginosa: evidence of iron override effects on quorum sensing and biofilm-specific gene regulation. J Bacteriol 183:1990–1996. doi:10.1128/JB.183.6.1990-1996.2001 11222597 PMC95094

[52] Pezzoni M, Meichtry M, Pizarro RA, Costa CS. 2015. Role of the Pseudomonas quinolone signal (PQS) in sensitising Pseudomonas aeruginosa to UVA radiation. J Photochem Photobiol B 142:129–140. doi:10.1016/j.jphotobiol.2014.11.014 25535873

[53] Azimi S, Klementiev AD, Whiteley M, Diggle SP. 2020. Bacterial quorum sensing during infection. Annu Rev Microbiol 74:201–219. doi:10.1146/annurev-micro-032020-093845 32660382 PMC13064819

[54] Lesic B, Lépine F, Déziel E, Zhang J, Zhang Q, Padfield K, Castonguay MH, Milot S, Stachel S, Tzika AA, Tompkins RG, Rahme LG. 2007. Inhibitors of pathogen intercellular signals as selective anti-infective compounds. PLoS Pathog 3:1229–1239. doi:10.1371/journal.ppat.0030126 17941706 PMC2323289

