# Supplementary material for: Laser desorption rapid evaporative ionization mass spectrometry (LD-REIMS) demonstrates a direct impact of hypochlorous acid stress on PQS-mediated quorum sensing in Pseudomonas aeruginosa
Source: mSystems. 2024 Mar 26;9(4):e01165-23. doi: 10.1128/msystems.01165-23 (PMC11019781; doi:10.1128/msystems.01165-23)

**SUPPLEMENTARY**

**
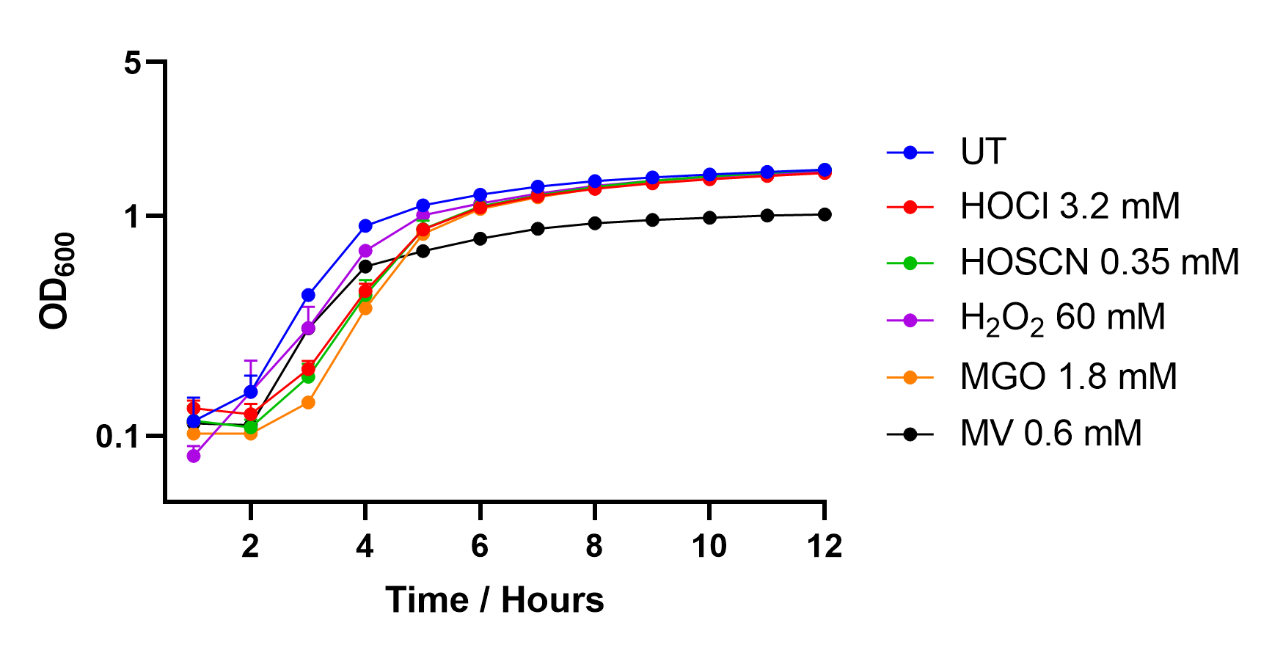
**

Figure S1: Growth data for untreated samples compared to samples treated with oxidants at the concentrations employed in this study. Bacteria were subcultured into untreated or treated LB in a 96-well plate format and incubated overnight at 37 °C, shaking at 700 rpm. Points represent the mean OD_600_ value at each timepoint, whilst the error bars represent the standard deviation of the mean, (n=6). A range of concentrations were tested, with the aim to find a concentration for each oxidant that provided a one-hour lag in growth compared to untreated. This criterion was chosen to ensure the bacteria were responding to the stress whilst not being killed.


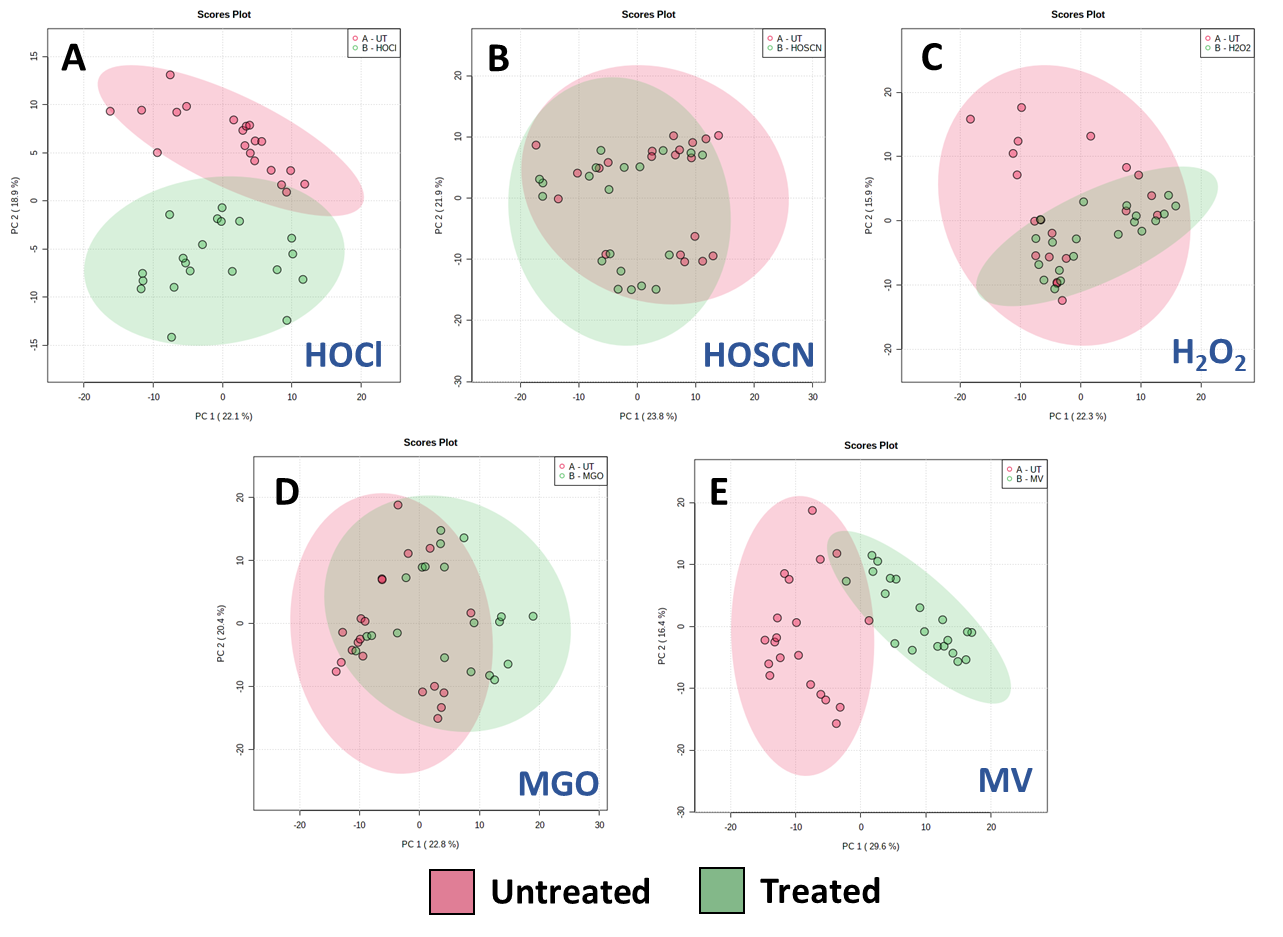


Figure S2: Statistical analysis of P. aeruginosa mass spectrum data obtained by LD-REIMS by a two-group PCA of untreated and treated P. aeruginosa samples; (a) 3.2 mM HOCl, (b) 0.35 mM HOSCN, (c) 60 mM H2O2; (d) 1.8 mM MGO (e) 0.6 mM MV. Samples were prepared and analysed over two independent days, n=20 (10 per day). Statistical analyses were completed on the 50 to 1200 m/z range, after background subtraction and mass drift correction, using the MetaboAnalyst 5.0 platform. The plots all represent components one and two. Shaded areas show 95% confidence intervals of the sample groups. The HOCl- and MV-treated samples (panels A and E respectively) clearly separate from the untreated, suggesting a strong impact of the metabolic profile. The samples for the other treatments (HOSCN, H_2_O_2_ and MGO, panels B, C and E respectively) do not clearly separate from untreated at the concentration used.


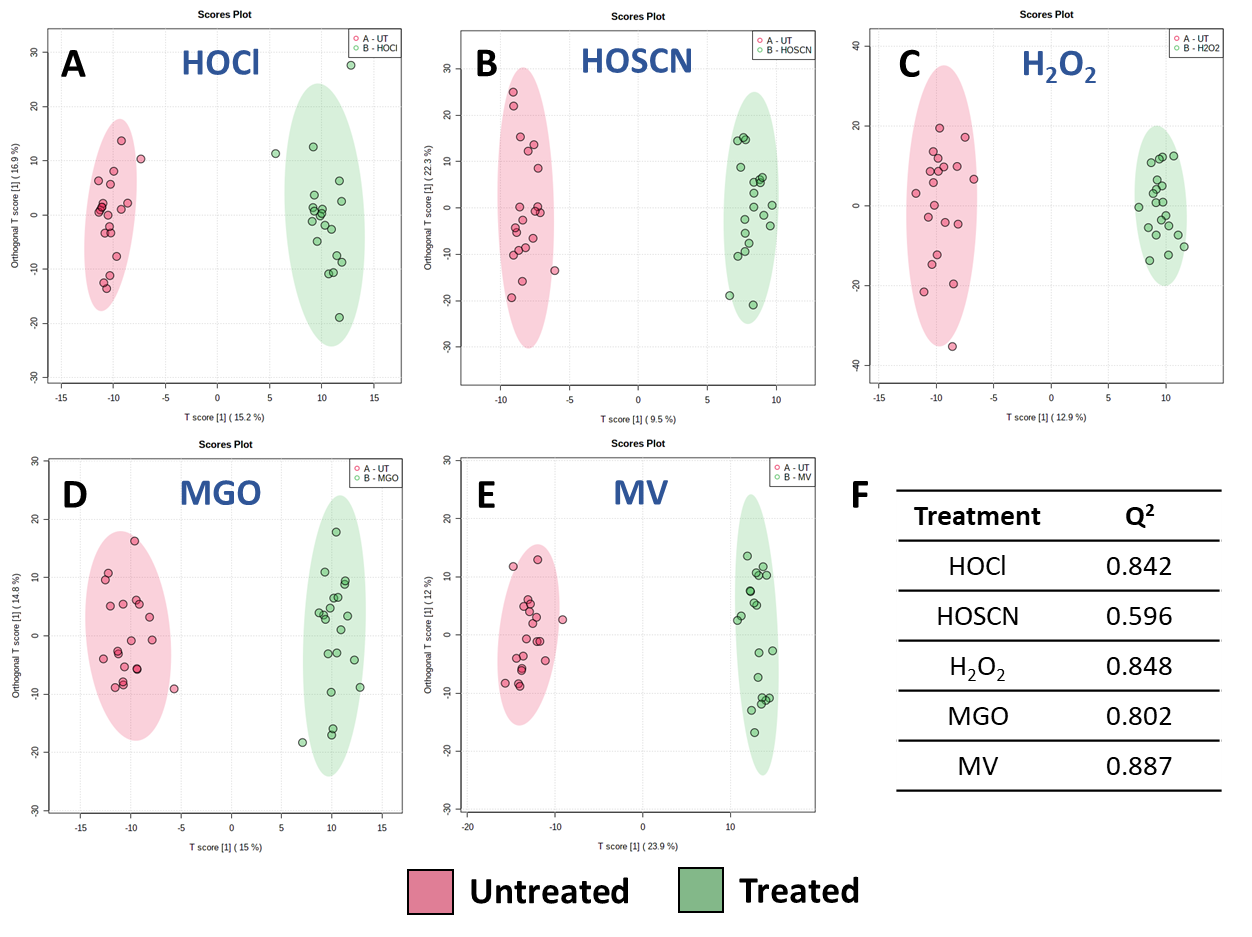


Figure S3: Statistical analysis of P. aeruginosa mass spectrum data obtained by LD-REIMS by OPLS-DA analysis. Score plots generated based on OPLS-DA of untreated and treated P. aeruginosa samples; (a) 3.2 mM HOCl, (b) 0.35 mM HOSCN, (c) 60 mM H2O2; (d) 1.8 mM MGO (e) 0.6 mM MV. Samples were prepared and analysed over two independent days, n=20 (10 per day). Statistical analyses were completed on the 50 to 1200 m/z range, after background subtraction and mass drift correction, using the MetaboAnalyst 5.0 platform. The model is able to separate the untreated and treated samples for each oxidant, cross-validated by the high Q^2^ coefficient for each plot (see table).


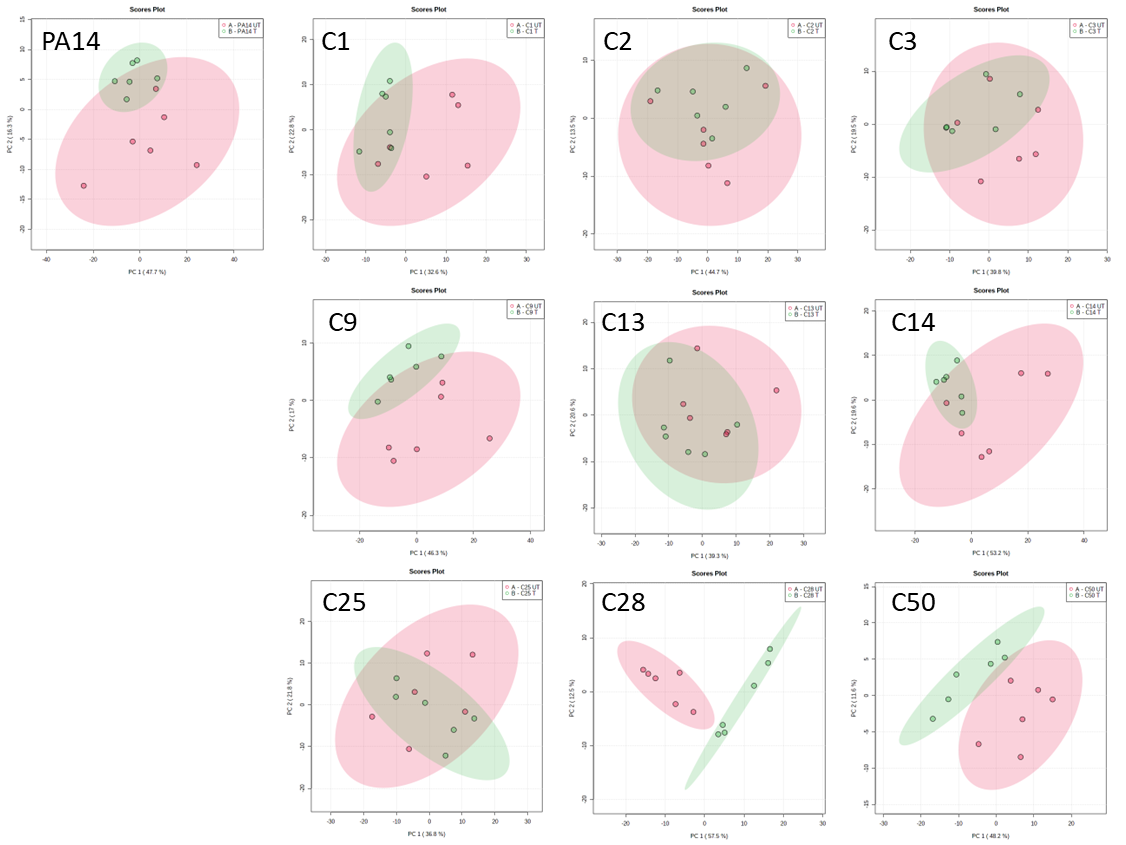


Figure S4: Two-group PCA of untreated and HOCl-treated P. aeruginosa strains, comparing PA14 to 10 cystic fibrosis (CF) isolates (n=6). Statistical analyses were completed on the 50 to 1200 m/z range, after background subtraction and mass drift correction, using the MetaboAnalyst 5.0 platform. Separation between untreated and treated samples to the same degree as PA14 is apparent for C1, C9, C13, C14, C15, C28 and C50, suggesting the response to HOCl is conserved in these strains.


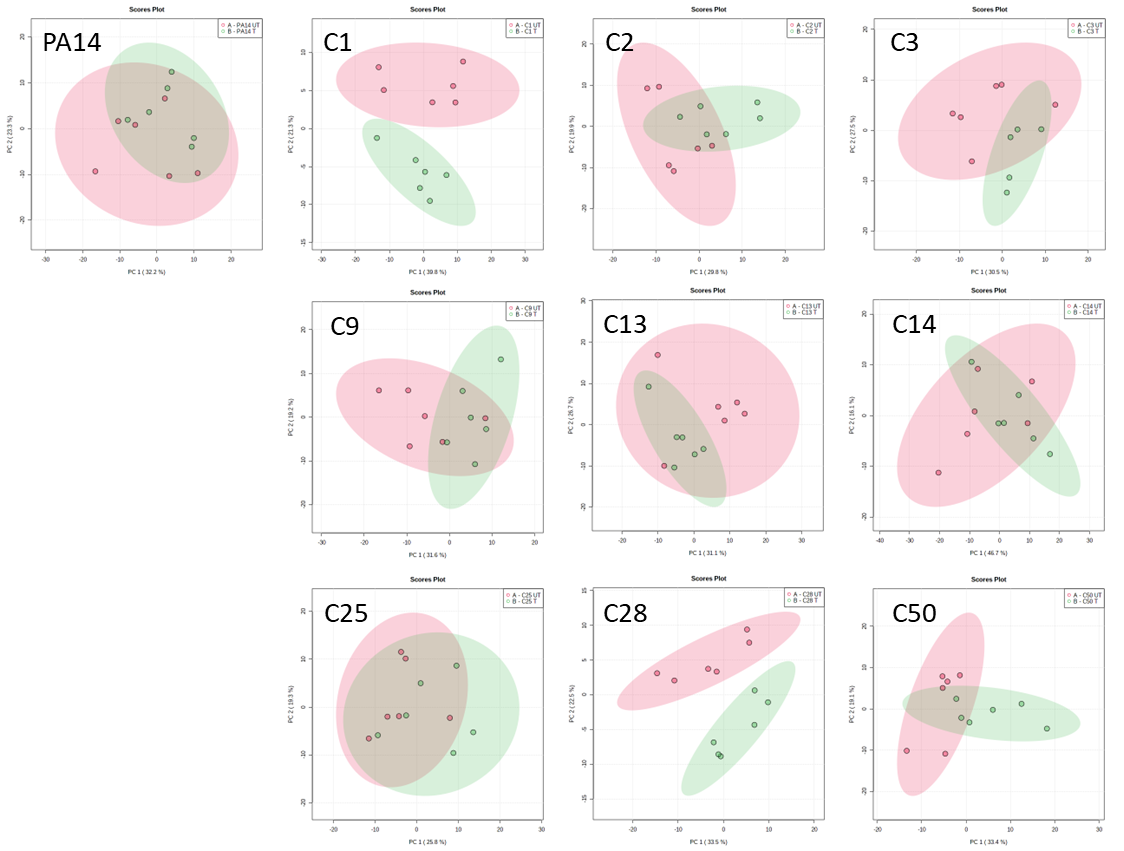


Figure S5: Two-group PCA of untreated and HOSCN-treated P. aeruginosa strains, comparing PA14 to 10 cystic fibrosis (CF) isolates (n=6). Statistical analyses were completed on the 50 to 1200 m/z range, after background subtraction and mass drift correction, using the MetaboAnalyst 5.0 platform. Separation between untreated and treated samples to the same degree as PA14

Figure S8: Pyocyanin concentrations in stationary phase P. aeruginosa cultures were not impacted by treatment with HOCl, suggesting the decrease in concentration seen in growing cultures exposed to HOCl is not due to direct oxidisation of pyocyanin.

Table S1: P. aeruginosa strains used in this study.

| **Strain** | **Description** | **Source** |
| --- | --- | --- |
| PA14 (WT) | Wild-type lab stock | M. Ausubel, Harvard (Rahme et al.*,* 1995) |
| *pqsE* | PAMr: PA14 wild-type background, MAR2xT7 transposon, Gm^r^. PqsE Quinolone signal response protein. | M. Ausubel, Harvard (Liberati NT et al., 2006) |
| C1 | *P. aeruginosa* clinical isolate from early infection of CF lung | Colindale |
| C2 | *P. aeruginosa* clinical isolate from early infection of CF lung | Colindale |
| C3 | *P. aeruginosa* clinical isolate from early infection of CF lung | Colindale |
| C9 | *P. aeruginosa* clinical isolate from early infection of CF lung | Colindale |
| C28 | *P. aeruginosa* clinical isolate from early infection of CF lung | Colindale |
| C50 | *P. aeruginosa* clinical isolate from early infection of CF lung | Colindale |
| C12 | *P. aeruginosa* clinical isolate from chronic infection of CF lung | Royal Brompton Hospital |
| C13 | *P. aeruginosa* clinical isolate from chronic infection of CF lung | Royal Brompton Hospital |
| C14 | *P. aeruginosa* clinical isolate from chronic infection of CF lung | Royal Brompton Hospital |
| C15 | *P. aeruginosa* clinical isolate from chronic infection of CF lung | Royal Brompton Hospital |
| C17 | *P. aeruginosa* clinical isolate from chronic infection of CF lung | Royal Brompton Hospital |
| C25 | *P. aeruginosa* clinical isolate from chronic infection of CF lung | Royal Brompton Hospital |

**All identified metabolites with concentration significantly impacted by exposure to one or more of the tested oxidants:**

Table S2: All metabolites significantly impacted by oxidant treatment identified to at least MSI3.

| **Annotation** | **Detected ion** | **Accurate m/z** | **Theoretical m/z** | **Error (ppm)** | **MSI** |
| --- | --- | --- | --- | --- | --- |
| C7:db-HHQ | [M-H]- | 240.1386 | 240.1394 | -3.331 | 3 |
| PQS HQNO | [M-H]- | 258.1493 | 258.1500 | -2.712 | 3 |
| C9:db-NHQ | [M-H]- | 268.1699 | 268.1707 | -2.983 | 3 |
| NHQ | [M-H]- | 270.1857 | 270.1863 | -2.221 | 2 |
| C9:db-PQS  C9:db-NQNO | [M-H]- | 284.1648 | 284.1656 | -2.815 | 3 |
| C9-PQS  C9-NQNO | [M-H]- | 286.1809 | 286.1813 | -1.398 | 3 |
| C11:db-UHQ | [M-H]- | 296.2010 | 296.2020 | -3.376 | 2 |
| UHQ | [M-H]- | 298.2173 | 298.2176 | -1.006 | 2 |
| C11:db-PQS  C11:db-UQNO | [M-H]- | 312.1958 | 312.1969 | -3.523 | 2 |
| C11-PQS  C11-UQNO | [M-H]- | 314.2105 | 314.2126 | -6.683 | 2 |
| Rha-C10 | [M-H]- | 333.1909 | 333.1914 | -1.501 | 3 |
| Rha-C10-C10-CH3  Rha-C11-C9-CH3 | [M-H]- | 517.3356 | 517.3377 | -4.059 | 3 |
| Rha-C10-C12:1 | [M-H]- | 529.3377 | 529.3377 | 0.000 | 3 |
| Rha-C10-C12 | [M-H]- | 531.3534 | 531.3534 | 0.000 | 2 |
| Rha-C10-C10 | [M+Cl]- | 539.2986 | 539.2987 | -0.185 | 3 |
| Rha-C8-C10-CH3 | [M-H]- | 545.3596 | 545.3690 | -17.236 | 3 |
| Rha-Rha-C10-C12:1 | [M-H]- | 675.3931 | 675.3956 | -3.702 | 3 |
| Rha-Rha-C10-C12 | [M-H]- | 677.4106 | 677.4113 | -1.033 | 2 |
| Rha-Rha-C12-C12 | [M-H]- | 705.4439 | 705.4431 | 1.134 | 3 |
| PE(30:0) | [M-H]- | 662.4751 | 662.4761 | -1.509 | 2 |
| PA(34:1) | [M-H]- | 673.4812 | 673.4814 | -0.297 | 3 |
| PE(32:2) | [M-H]- | 686.4746 | 686.4766 | -2.913 | 3 |
| PE(32:1) | [M-H]- | 688.4902 | 688.4923 | -3.050 | 2 |
| PE(32:0) | [M-H]- | 690.5051 | 690.5079 | -4.055 | 2 |
| PG(30:0) | [M-H]- | 693.4734 | 693.4707 | 3.893 | 2 |
| PE(33:2) | [M-H]- | 700.4902 | 700.4918 | -2.284 | 3 |
| PE(33:1) | [M-H]- | 702.5056 | 702.5074 | -2.562 | 2 |
| PE(33:0) | [M-H]- | 704.5186 | 704.5231 | -6.387 | 2 |
| PE(34:2) | [M-H]- | 714.5062 | 714.5079 | -2.379 | 3 |
| PE(34:1) | [M-H]- | 716.5231 | 716.5231 | 0.000 | 2 |
| PE(34:0) | [M-H]- | 718.5305 | 718.5387 | -11.412 | 3 |
| PG(32:1) | [M-H]- | 719.4958 | 719.4868 | 12.509 | 2 |
| PG(32:0) | [M-H]- | 721.4997 | 721.5020 | -3.188 | 2 |
| PE(35:2) | [M-H]- | 728.5221 | 728.5236 | -2.059 | 3 |
| PE(35:1) | [M-H]- | 730.5365 | 730.5392 | -3.696 | 2 |
| PG(33:1) | [M-H]- | 733.5014 | 733.5020 | -0.818 | 2 |
| PG(33:0) | [M-H]- | 735.5128 | 735.5177 | -6.662 | 2 |
| PE(36:2) | [M-H]- | 742.5399 | 742.5392 | 0.943 | 3 |
| PE(36:1) | [M-H]- | 744.5514 | 744.5544 | -4.029 | 3 |
| PG(34:2) | [M-H]- | 745.5013 | 745.5025 | -1.610 | 2 |
| PG(34:1) | [M-H]- | 747.5173 | 747.5181 | -1.070 | 3 |
| PG(34:0) | [M-H]- | 749.5251 | 749.5244 | 0.934 | 3 |
| PA(40:4) | [M-H]- | 751.5321 | 751.5278 | 5.722 | 3 |
| PG(35:2) | [M-H]- | 759.5166 | 759.5181 | -1.975 | 2 |
| PG(35:1) | [M-H]- | 761.5314 | 761.5333 | -2.495 | 2 |
| PG(35:0) | [M-H]- | 763.5314 | 763.5283 | 4.060 | 2 |
| PS(35:3) | [M-H]- | 770.4961 | 770.4973 | -1.557 | 3 |
| PG(36:2) | [M-H]- | 773.5316 | 773.5338 | -2.844 | 3 |
| PS(37:3) | [M-H]- | 798.5255 | 798.5286 | -3.882 | 3 |
| PS(38:0) | [M-H]- | 818.5872 | 818.5912 | -4.886 | 3 |
| PG(40:2) | [M-H]- | 829.5879 | 829.5959 | -9.643 | 3 |
| PS(39:1) | [M-H]- | 830.5886 | 830.5912 | -3.130 | 3 |
| PS(39:0) | [M-H]- | 832.6060 | 832.6068 | -0.961 | 3 |
| PG(43:6) | [M-H]- | 863.5857 | 863.5830 | 3.127 | 3 |

Table S3: Identification of 24 of the most significant molecules based on MSMS spectral data acquired with LCMS analysis. The four most intense fragment ions are given together their parent ion exact mass. Intensities are given related to the most intense fragment ion. Unattributed fragment ions were included in the table; isotope ions were excluded.

| **Annotation** | **Theoretical m/z** | **Error (ppm)** | **Four main fragments m/z (intensity relative to the most abundant fragment)** | | | | **Parent ion** |
| --- | --- | --- | --- | --- | --- | --- | --- |
| NHQ | 270.1863 | -2.221 | 143.0 (3%) | 157.1 (23%) | 158.1 (4%) | 170.1 (7%) | 270.2 (100%) |
| C11:db-UHQ | 296.202 | -3.376 | 143.0 (7%) | 157.1 (17%) | 158.1 (59%) | 170.1 (8%) | 296.2 (100%) |
| UHQ | 298.2176 | -1.006 | 157.1 (9%) | 199.1 (5%) | 280.2 (9%) | 297.2 (20%) | 298.2 (100%) |
| C11:db-PQS  C11:db-UQNO | 312.1969 | -3.523 | 144.0 (33%) | 159.0 (9%) | 170.1 (100%) | 294.2 (18%) | 312.2 (51%) |
| C11-PQS  C11-UQNO | 314.2126 | -6.683 | 97.0 (26%) | 198.9 (24%) | 296.2 (23%) | 313.2 (25%) | 314.2 (100%) |
| Rha-C10-C12 | 531.3534 | 0.000 | 101.0 (11%) | 169.1 (100%) | 197.2 (48%) | 333.2 (28%) | 531.4 (14%) |
| Rha-Rha-C10-C12 | 677.4113 | 0.000 | 169.1 (49%) | 197.2 (24%) | 205.1 (32%) | 479.2 (73%) | 677.4 (100%) |
| PE(30:0) | 662.4761 | -1.509 | 241.2 (100%) | 227.2 (14%) | 255.2 (9%) | 438.3 (2%) | 662.5 (8%) |
| PE(32:1) | 688.4923 | -3.050 | 227.2 (100%) | 281.2 (43%) | 255.2 (15%) | 253.2 (12%) | 688.5 (8%) |
| PE(32:0) | 690.5079 | -4.055 | 255.2 (100%) | 452.3 (2%) | 140.0 (1%) | 196.0 (1%) | 690.5 (5%) |
| PG(30:0) | 693.4707 | 3.893 | 241.2 (100%) | 153.0 (3%) | 377.2 (1%) | 469.3 (1%) | 693.5 (16%) |
| PE(33:1) | 702.5074 | -2.562 | 241.2 (62%) | 253.2 (9%) | 255.2 (13%) | 281.2 (24%) | 702.5 (100%) |
| PE(33:0) | 704.5231 | -6.387 | 241.2 (7%) | 255.2 (76%) | 269.2 (38%) | 466.3 (5%) | 704.5 (100%) |
| PE(34:1) | 716.5231 | 0.000 | 255.2 (100%) | 281.2 (40%) | 478.3 (2%) | 140.0 (1%) | 716.5 (9%) |
| PG(32:1) | 719.4868 | 12.509 | 227.2 (100%) | 281.2 | 253.2 | 255.2 | 719.5 |
| PG(32:0) | 721.502 | -3.188 | 255.2 (100%) | 153.0 (4%) | 391.2 (2%) | 483.3 (2%) | 721.5 (23%) |
| PE(35:1) | 730.5392 | -3.696 | 269.2 (100%) | 281.2 (41%) | 255.2 (9%) | 478.3 (3%) | 730.5 (15%) |
| PG(33:1) | 733.502 | -0.818 | 241.2 (100%) | 281.2 (50%) | 255.2 (17%) | 253.2 (12%) | 733.5 (40%) |
| PG(33:0) | 735.5177 | -6.662 | 241.2 (3%) | 255.2 (33%) | 269.2 (16%) | 405.2 (2%) | 735.5 (100%) |
| PG(34:2) | 745.5025 | -1.610 | 253.2 (100%) | 281.2 (36%) | 153.0 (5%) | 417.2 (2%) | 745.5 (32%) |
| PG(35:2) | 759.5181 | -1.975 | 253.2 (11%) | 267.2 (25%) | 281.2 (12%) | 295.3 (5%) | 759.5 (100%) |
| PG(35:1) | 761.5333 | -2.495 | 269.2 (100%) | 281.2 (51%) | 255.2 (25%) | 241.2 (16%) | 761.5 (46%) |
| PG(35:0) | 763.5283 | 4.060 | 269.2 (31%) | 405.2 (20%) | 676.5 (44%) | 762.5 (16%) | 763.5 (100%) |

Table S4: Fold change in intensity and the associated p value for each identified metabolite when exposed to each oxidant. P values were calculated using Students t-test, with a threshold of 0.05. Fold change is calculated using the absolute values of change between the two group means, prior to normalisation.

| **Annotation** | **Class** | **HOCl** |  | **HOSCN** |  | **H_2_O_2_** |  | **MGO** |  | **MV** |  |
| --- | --- | --- | --- | --- | --- | --- | --- | --- | --- | --- | --- |
|  |  | **log_10_*(p)*** | **log_2_(FC)** | **log_10_*(p)*** | **log_2_(FC)** | **log_10_*(p)*** | **log_2_(FC)** | **log_10_*(p)*** | **log_2_(FC)** | **log_10_*(p)*** | **log_2_(FC)** |
| C7:db-HHQ | QSM | -3.89 | -1.98 |  |  |  |  | -2.58 | 0.84 |  |  |
| PQS  HQNO | QSM | -6.03 | -1.25 |  |  |  |  | -2.23 | 0.48 | -3.93 | -0.85 |
| C9:db-NHQ | QSM | -12.32 | -3.06 |  |  |  |  |  |  |  |  |
| NHQ | QSM | -9.70 | -2.34 |  |  |  |  |  |  |  |  |
| C9:db-PQS C9:db-NQNO | QSM | -8.18 | -1.51 |  |  | -3.16 | -0.77 |  |  |  |  |
| C9-PQS C9-NQNO | QSM | -3.28 | -0.87 |  |  |  |  |  |  |  |  |
| C11:db-UHQ | QSM | -12.26 | -2.73 |  |  |  |  |  |  | -3.49 | -1.14 |
| UHQ | QSM | -9.93 | -3.25 |  |  |  |  |  |  | -6.59 | -1.39 |
| C11:db-PQS C11:db-UQNO | QSM | -4.43 | -1.17 |  |  |  |  |  |  |  |  |
| C11-PQS C11-UQNO | QSM | -5.87 | -1.37 |  |  |  |  |  |  | -2.44 | -0.77 |
| Rha-C10 | RL |  |  |  |  | -5.96 | -1.21 | -2.23 | -0.64 |  |  |
| Rha-C10-C10-CH3 Rha-C11-C9-CH3 | RL | -3.48 | -0.92 |  |  | -10.47 | -2.02 | -6.26 | -1.36 |  |  |
| Rha-C10-C12:1 | RL |  |  |  |  | -8.63 | -1.73 | -3.69 | -1.03 |  |  |
| Rha-C10-C12 | RL |  |  |  |  | -8.83 | -2.14 | -4.50 | -1.38 |  |  |
| Rha-C10-C10 | RL |  |  |  |  | -2.33 | -0.90 |  |  | -3.29 | 0.67 |
| Rha-C8-C10-CH3 | RL | -3.64 | -1.35 |  |  | -4.56 | -1.68 |  |  |  |  |
| Rha-Rha-C10-C12:1 | RL |  |  |  |  | -6.13 | -2.28 |  |  |  |  |
| Rha-Rha-C10-C12 | RL |  |  |  |  | -7.97 | -2.25 | -2.63 | -1.11 |  |  |
| Rha-Rha-C12-C12 | RL | -2.43 | -0.88 |  |  | -8.18 | -2.88 | -4.19 | -1.68 |  |  |
| PE(30:0) | PE | -8.04 | 0.61 | -16.08 | 1.24 | 2.70 | 0.31 | 12.65 | 0.83 |  |  |
| PA(34:1) | PA | -3.45 | -0.92 | -2.25 | -0.72 |  |  | 3.49 | -0.90 | 2.27 | -0.71 |
| PE(32:2) | PE | -2.85 | 0.74 | -4.46 | 1.04 | 2.97 | 0.77 | 3.17 | 0.76 | 2.15 | 0.54 |
| PE(32:1) | PE | -7.72 | 0.66 | -14.30 | 1.03 | 4.31 | 0.51 | 9.83 | 0.70 |  |  |
| PE(32:0) | PE | -5.90 | 0.50 | -12.79 | 0.96 |  |  | 8.82 | 0.68 | 4.43 | 0.50 |
| PG(30:0) | PG | -7.22 | 1.77 | -9.25 | 2.44 | 2.49 | 1.23 | 7.59 | 1.86 |  |  |
| PE(33:2) | PE |  |  |  |  |  |  |  |  | 6.92 | 1.27 |
| PE(33:1) | PE |  |  |  |  |  |  |  |  | 9.91 | 0.81 |
| PE(33:0) | PE |  |  |  |  |  |  |  |  | 7.93 | 0.86 |
| PE(34:2) | PE | -5.51 | 0.43 | -5.44 | 0.41 | 3.00 | 0.34 | 4.07 | 0.34 | 8.11 | 0.55 |
| PE(34:1) | PE |  |  |  |  |  |  |  |  | 4.28 | 0.29 |
| PG(32:1) | PG | -8.30 | 0.76 | -10.40 | 1.03 | 6.76 | 0.68 | 9.23 | 0.72 | 9.47 | -1.04 |
| PG(32:0) | PG | -6.64 | 0.50 | -15.02 | 1.06 |  |  | 10.05 | 0.59 | 1.58 | -0.17 |
| PE(35:2) | PE | -5.76 | -0.38 | -3.87 | -0.29 | 10.68 | -0.75 | 15.42 | -0.89 | 26.56 | 2.76 |
| PE(35:1) | PE | -6.02 | -0.48 | -8.14 | -0.62 | 7.40 | -0.63 | 9.51 | -0.69 | 18.23 | 1.97 |
| PG(33:1) | PG |  |  |  |  | 3.80 | -0.35 | 7.95 | -0.53 | 10.38 | 0.71 |
| PG(33:0) | PG |  |  |  |  | 2.47 | -0.25 | 3.27 | -0.28 | 5.51 | 0.43 |
| PE(36:2) | PE |  |  |  |  | 1.98 | 0.32 |  |  |  |  |
| PE(36:1) | PE |  |  |  |  |  |  | 2.65 | -0.27 |  |  |
| PG(34:2) | PG | -5.01 | 0.49 |  |  | 5.48 | 0.50 | 4.04 | 0.38 | 3.83 | -0.43 |
| PG(34:1) | PG |  |  |  |  |  |  |  |  | 8.60 | -0.56 |
| PG(34:0) | PG |  |  | -4.09 | -0.33 |  |  | 6.54 | -0.36 | 17.13 | -0.96 |
| PA(40:4) | PA |  |  | -5.98 | -0.44 |  |  | 6.09 | -0.38 | 8.20 | -0.53 |
| PG(35:2) | PG | -10.00 | -0.81 | -3.99 | -0.48 | 16.67 | -1.38 | 18.89 | -1.48 | 28.09 | 2.76 |
| PG(32:1) | PG | -14.65 | -1.14 | -10.07 | -0.94 | 20.18 | -1.51 | 23.06 | -1.62 | 21.74 | 2.01 |
| PG(35:0) | PG | -9.95 | -0.74 | -6.54 | -0.67 | 13.91 | -1.00 | 17.07 | -1.21 | 19.89 | 1.62 |
| PS(35:3) | PS | -5.39 | 3.59 | -8.46 | 4.23 | 4.20 | 2.97 | 6.21 | 3.63 | 6.62 | 2.73 |
| PG(36:2) | PG |  |  |  |  | 3.14 | 0.29 | 1.81 | -0.21 | 3.51 | -0.30 |
| PS(38:0) | PS | -2.44 | -0.47 | -2.29 | -0.45 | 3.23 | -0.66 | 4.02 | -0.69 |  |  |
| PG(40:2) | PG | -2.13 | 1.65 |  |  | 2.71 | 1.59 | 3.19 | 1.92 |  |  |
| PS(39:1) | PS | -3.82 | 1.52 |  |  | 3.71 | 1.41 | 5.37 | 1.80 | 1.51 | 0.29 |
| PS(39:0) | PS |  |  |  |  |  |  |  |  | 8.43 | -1.86 |
| PG(43:6) | PG |  |  |  |  |  |  |  |  | 1.85 | 0.69 |

Table S5: Fold change in intensity and the associated p value for each of the HOCl biomarkers established in PA14 in clinical isolates grown in HOCl. P values were calculated using Students t-test, with a threshold of 0.05. Fold change was calculated using the absolute values of change between the two group means, prior to normalisation.


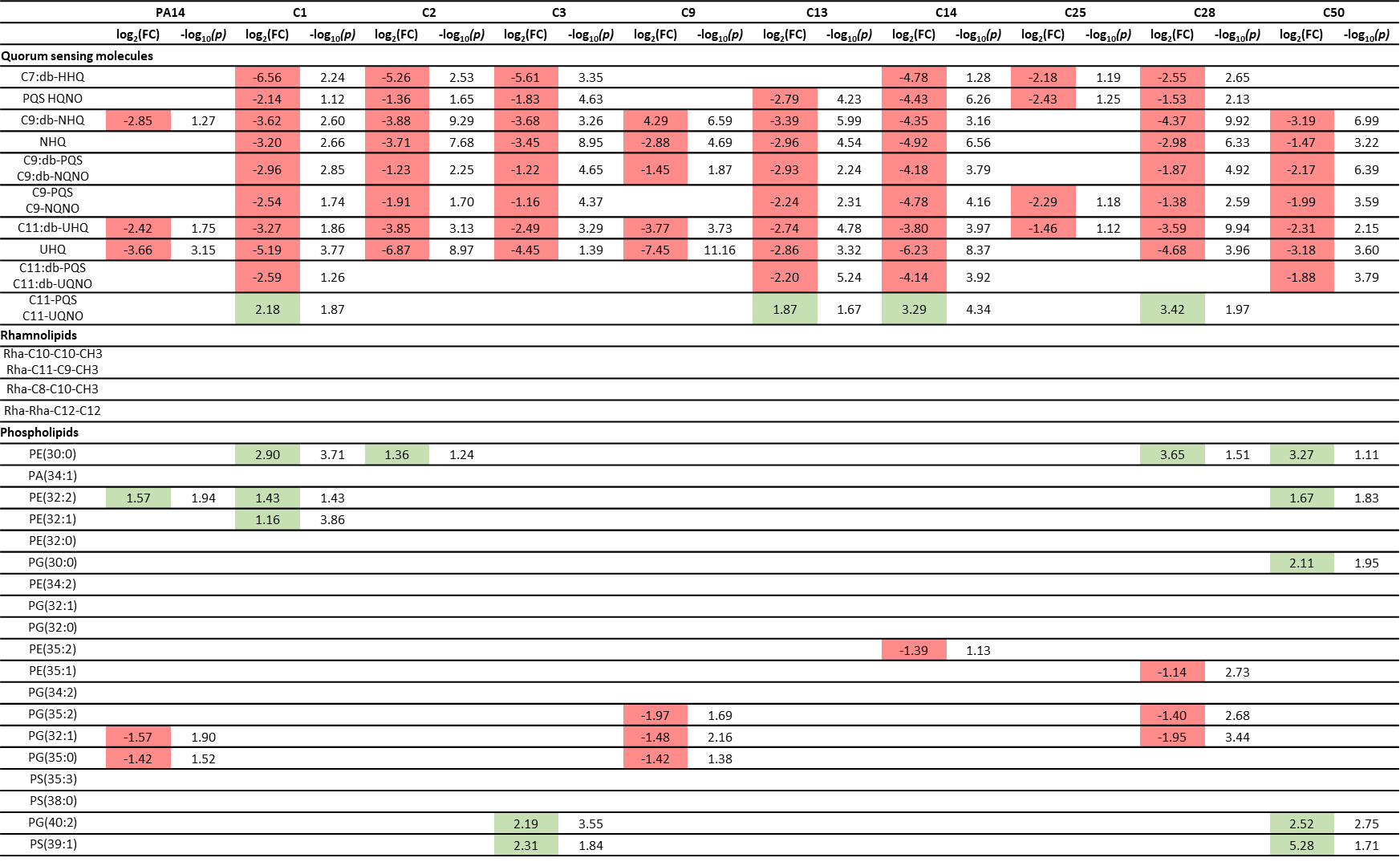


Table S6: Fold change in intensity and the associated p value for each of the HOSCN biomarkers established in PA14 in clinical isolates grown in HOSCN. P values were calculated using Students t-test, with a threshold of 0.05. Fold change was calculated using the absolute values of change between the two group means, prior to normalisation.


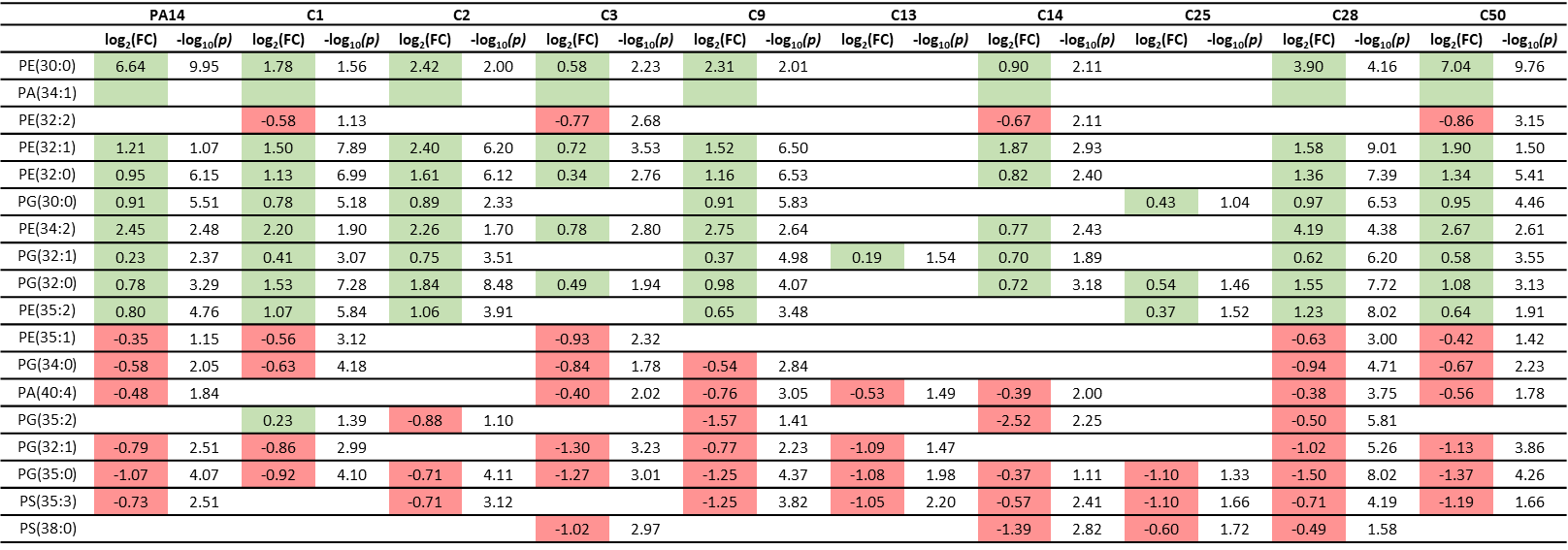

Supplement: Supplemental figures and tables — Figures S1 to S8 and Tables S1 to S6. [file msystems.01165-23-s0001.docx]
